# Rice Husk Ash Geopolymers Modified with Fe_3_O_4_ or ZnTiO_3_/TiO_2_ Nanoparticles for the Adsorption and Photodegradation of Organic Dyes

**DOI:** 10.3390/nano16070413

**Published:** 2026-03-29

**Authors:** Ximena Jaramillo-Fierro, Juan-Pablo Cueva, John Ramón, Eduardo Valarezo

**Affiliations:** 1 Departamento de Química, Facultad de Ciencias Exactas y Naturales, Universidad Técnica Particular de Loja, San Cayetano Alto, Loja 1101608, Ecuador; bevalarezo@utpl.edu.ec; 2 Carrera de Ingeniería Química, Facultad de Ciencias Exactas y Naturales, Universidad Técnica Particular de Loja, San Cayetano Alto, Loja 1101608, Ecuador; jpcueva13@utpl.edu.ec (J.-P.C.); jbramon2@utpl.edu.ec (J.R.)

**Keywords:** nanoparticles, ZnTiO_3_/TiO_2_, Fe_3_O_4_, geopolymer, rice husk ash, adsorption, photocatalysis, dye removal

## Abstract

Hybrid nanomaterials integrating magnetic and semiconductor phases offer promising multifunctional platforms for wastewater remediation; however, their stabilization and recovery remain challenging. In this study, Fe_3_O_4_ and ZnTiO_3_/TiO_2_ nanoparticles were incorporated into a rice husk ash-based geopolymer matrix to develop hybrid nanocomposites for synergistic adsorption–photodegradation of methylene blue (MB) and methyl orange (MO). The materials were synthesized via alkaline activation followed by nanoparticle incorporation, and characterized by XRD, XRF, FTIR, SEM, EDX, BET surface area analysis, and pHPZC determination. XRD confirmed the presence of nanocrystalline Fe_3_O_4_ and ZnTiO_3_/TiO_2_ phases while preserving the amorphous aluminosilicate framework. Modified powders exhibited higher specific surface areas (up to 198 m^2^ g^−1^) compared to the unmodified geopolymer. Adsorption followed the Langmuir isotherm and pseudo-second-order kinetics, with spontaneous and exothermic behavior. Under UV irradiation, the ZnTiO_3_/TiO_2_-modified composite achieved photodegradation efficiencies up to 94% for MB and 92% for MO, whereas the Fe_3_O_4_-modified material combined adsorption capacity with magnetic recoverability. These results demonstrate that nanoparticle incorporation enables multifunctional performance while maintaining structural integrity of the geopolymeric matrix.

## 1. Introduction

Recent reviews have highlighted the rapid development of nanostructured photocatalysts and magnetic nanomaterials for wastewater treatment due to their high surface reactivity and ability to promote pollutant degradation and adsorption processes [1,2]. The exceptional performance of these materials arises from their high surface-to-volume ratio, which increases the density of reactive sites available for pollutant capture and degradation [3]. At the nanoscopic scale, materials exhibit unique size-dependent properties that enhance interfacial charge transfer and light absorption, enabling superior photocatalytic kinetics compared to large-scale materials [4]. However, practical deployment of nanoparticle-based systems faces critical challenges: magnetic nanoparticles are susceptible to agglomeration under applied fields, reducing their effective surface area and dispersibility [5], while the absence of structural support complicates solid–liquid separation and limits reusability in continuous treatment cycles [6]. These limitations underscore the need for engineered architectures that preserve nanoscale functionality while ensuring operational stability and recoverability.

Within the family of nanomaterials, magnetite (Fe_3_O_4_) nanoparticles and ZnTiO_3_/TiO_2_ heterostructures represent complementary functional nano-phases that address distinct aspects of the remediation process. Fe_3_O_4_ nanoparticles exhibit superparamagnetic behavior at crystallite sizes below 50 nm, enabling efficient magnetic recovery without permanent aggregation [7]. The hydroxylated surface of magnetite (Fe–OH groups) provides abundant coordination sites for pollutant adsorption and can be chemically functionalized to enhance colloidal stability and selectivity [8]. Crucially, the magnetic response of Fe_3_O_4_ facilitates rapid solid–liquid separation, reducing processing time and enabling catalyst reuse—a prerequisite for economically viable water treatment [9,10]. Indeed, recent studies highlight the versatility of Fe_3_O_4_ nanoparticles in contaminant adsorption and catalytic processes in aqueous systems [11].

In parallel, ZnTiO_3_/TiO_2_ heterostructures leverage band alignment at the perovskite–oxide interface to enhance photocatalytic efficiency. The type-II heterojunction formed between ZnTiO_3_ (band gap ~3.2 eV) and anatase TiO_2_ (band gap ~3.2 eV) promotes spatial separation of photogenerated electrons and holes: electrons migrate preferentially to TiO_2_ while holes accumulate in ZnTiO_3_, reducing recombination rates and increasing the population of charge carriers available for surface reactions [12,13,14,15]. Experimental studies report kinetic rate constants for ZnTiO_3_/TiO_2_ composites up to 3.7 times higher than those of bare TiO_2_ under solar irradiation, attributed to enhanced interfacial charge transfer and improved light absorption [16]. Furthermore, the porous morphology achievable in ZnTiO_3_/TiO_2_ heterostructures increases the surface area accessible to pollutants, creating a synergistic coupling between adsorption and photocatalytic degradation [17]. These materials are thus best understood not as isolated compounds but as integrated nano-phases within multifunctional composites. Titanium oxides, including TiO_2_ and ZnTiO_3_, remain among the most researched photocatalysts due to their chemical stability, high oxidative potential, and environmental compatibility. Consequently, numerous review studies have summarized recent advances in TiO_2_- and ZnTiO_3_-based photocatalysis for the degradation of organic pollutants in aquatic systems [18,19].

Despite their functional advantages, unsupported nanoparticles suffer from intrinsic stability problems that compromise long-term performance. Agglomeration driven by van der Waals forces and magnetic dipole interactions reduces the effective surface area and blocks active sites, particularly under the magnetic fields required for separation [20]. Recovery from dilute suspensions remains inefficient without magnetic cores or surface modifications, and repeated use leads to mechanical attrition and loss of nanomaterial into treated effluents [21]. Activity loss may occur through several pathways, including nanoparticle sintering during high-temperature processing, leaching of active phases into solution, and phase transformations such as the anatase-to-rutile transition in TiO_2_ above 600 °C, which increases charge recombination rates and reduces photocatalytic activity [22].

To overcome these limitations, recent strategies have focused on immobilization within structured inorganic matrices that provide mechanical support, prevent particle aggregation, and enhance thermal stability [23,24]. Embedding nanoparticles in porous hosts maintains high surface accessibility while anchoring active phases against leaching and sintering. Successful examples include TiO_2_ supported on industrial waste substrates, which demonstrated enhanced dispersion and adsorption–photocatalysis synergy with efficiencies up to 4.4-fold higher than unsupported TiO_2_ [25]. Such approaches highlight the critical role of the host matrix in preserving nanoscale functionality while enabling practical handling and reuse.

Geopolymers—amorphous aluminosilicate networks formed through alkali activation of Si- and Al-rich precursors—have emerged as promising nano-architectured supports for photocatalytic nanoparticles [26,27,28]. The mesoporous structure facilitates pollutant diffusion to embedded nanoparticles while the rigid inorganic framework prevents sintering and leaching, addressing the stability challenges inherent to unsupported nanomaterials. Geopolymers therefore act not only as structural binders but also as nanostructured hosts that modulate interfacial interactions and enhance overall system performance. The structural versatility of geopolymers strongly depends on the nature of the precursor employed. Therefore, the selection of sustainable and silica-rich raw materials is a key factor in designing geopolymer-based hybrid nanocomposites. Rice husk ash (RHA), an abundant agricultural by-product generated during rice production, is particularly attractive due to its high amorphous silica content and availability at low cost [29]. RHA-based geopolymers exhibit well-developed aluminosilicate networks, adjustable porosity, and favorable surface properties, making them suitable supports for functional fillers [30]. The valorization of agricultural waste in polymer synthesis further aligns geopolymer research with circular and green polymer science principles.

Unlike conventional ceramic binders, geopolymers develop a three-dimensional nano-network of [SiO_4_]^4−^ and [AlO_4_]^5−^ tetrahedra linked by shared oxygen atoms, creating a mesoporous scaffold with interconnected pores in the 2–50 nm range [31]. This nano-network provides to the structure high chemical stability, mechanical strength, thermal resistance, and a reduced carbon footprint compared to traditional cementitious materials [32]. Additionally, the porous architecture of the geopolymers provides high surface density of silanol (Si–OH) and aluminol (Al–OH) groups that facilitate chemical anchoring of metal oxide nanoparticles through condensation reactions, ensuring uniform dispersion and strong interfacial bonding [33,34]. The net negative charge of the aluminosilicate framework further promotes electrostatic adsorption of cationic pollutants, adding a complementary capture mechanism to photocatalytic degradation [35,36].

Among the most pressing environmental challenges addressed by functional polymeric materials is the contamination of water resources by synthetic dyes. Dyes are extensively used in textiles, paper, leather, food, and pharmaceutical industries, and a significant fraction is discharged into wastewater streams during industrial processing [37]. These compounds are particularly problematic due to their complex aromatic structures, high chemical stability, and resistance to biodegradation [38]. Among synthetic dyes, methylene blue (MB) and methyl orange (MO) are widely used in industrial processes [39]. MB is a cationic dye extensively used in textile, paper, and leather industries and is considered a hazardous pollutant due to its high solubility and toxicity [40]. Its discharge into aquatic systems increases turbidity, reduces light penetration, and limits dissolved oxygen, disrupting photosynthetic processes and aquatic organisms and [41]. MO is an anionic azo dye widely applied in textile printing and other industrial sectors, is characterized by high color intensity and poor biodegradability, which hinder its removal by conventional treatment methods [42]. Similar to MB, MO negatively impacts aquatic ecosystems even at low concentration and poses risks to animal and human health upon prolonged exposure. Due to their structural stability and persistence, both dyes remain challenging pollutants in wastewater treatment systems [43].

Multiple technologies have been developed to remove dyes from aqueous media, including biological, chemical, and physicochemical processes [44]. However, conventional methods such as coagulation–flocculation, membrane filtration, and ion exchange often suffer from incomplete removal efficiency, high operational costs, and secondary waste generation [45]. Among available approaches, adsorption has been widely recognized as one of the most effective and operationally simple methods for dye removal, particularly when high-surface-area materials with tunable surface chemistry are employed [46]. Geopolymers exhibit intrinsic adsorption capacity due to their negatively charged aluminosilicate framework, ion-exchange properties, and adjustable porosity, making them attractive inorganic polymer adsorbents [47]. Compared to conventional organic polymers and natural adsorbents, geopolymers offer superior thermal and chemical stability, enabling regeneration and reuse without significant degradation of performance [48]. In parallel with adsorption technologies, advanced oxidation processes (AOPs) have gained significant attention as effective strategies for the degradation of persistent organic pollutants [49]. Thus, the integration of semiconductor heterostructures into polymeric or inorganic polymer matrices provides a pathway toward hybrid materials that combine adsorption and photocatalytic functionalities within a single multifunctional composite.

Although several studies have reported the use of geopolymer-based materials for dye adsorption or photocatalysis individually [50], important knowledge gaps remain: (i) effect of the incorporation of crystalline magnetic and semiconductor phases in the amorphous geopolymer nano-network; (ii) quantitative elucidation of adsorption–photocatalysis synergy mechanisms in confined environments; and (iii) long-term recoverability and stability under realistic reuse conditions. Addressing these gaps requires integrated experimental approaches combining structural characterization (to confirm chemical composition and phase preservation) and performance evaluation under cyclic operation (to validate practical durability). This information is essential for the rational design of multifunctional geopolymer composites capable of coupling adsorption and photocatalytic degradation mechanisms within a single material platform.

Although magnetic nanoparticles and TiO_2_-based photocatalysts have been extensively studied for environmental remediation, the integration of different functional nanophases within structured geopolymeric matrices derived from agricultural waste remains comparatively less explored. In particular, rice husk ash-based geopolymers offer a sustainable platform capable of stabilizing nanoparticles while simultaneously providing adsorption capacity and mechanical integrity. In addition to nanoparticle incorporation, the materials developed in this study were engineered in pelletized form. This structural configuration facilitates the recovery of the adsorbent/catalyst after treatment, improves solid–liquid separation, and enables repeated reuse cycles without the need for complex separation techniques. Such an approach addresses an important limitation of nanoparticle-based remediation systems, where catalyst recovery from aqueous media remains challenging.

Therefore, the novelty of this work lies not only in incorporating Fe_3_O_4_ and ZnTiO_3_/TiO_2_ nanoparticles into a rice husk ash-based geopolymer matrix, but also in developing a structured multifunctional material that integrates adsorption, photocatalysis, and catalyst recoverability within a single platform. The materials were synthesized and comparatively evaluated in terms of their structural, morphological, and physicochemical properties, as well as their performance in the removal of methylene blue and methyl orange from aqueous solutions. Particular attention is given to the influence of magnetic and semiconductor nanophases on adsorption and photocatalytic behavior, aiming to establish structure–performance relationships. The reusability of the modified geopolymers over multiple treatment cycles was also assessed to examine their operational stability. Through this comparative approach, the study aims to clarify how different nanoparticle types influence the multifunctional performance of rice husk ash-derived geopolymer matrices for wastewater treatment applications.

## 2. Materials and Methods

**Figure 1:**
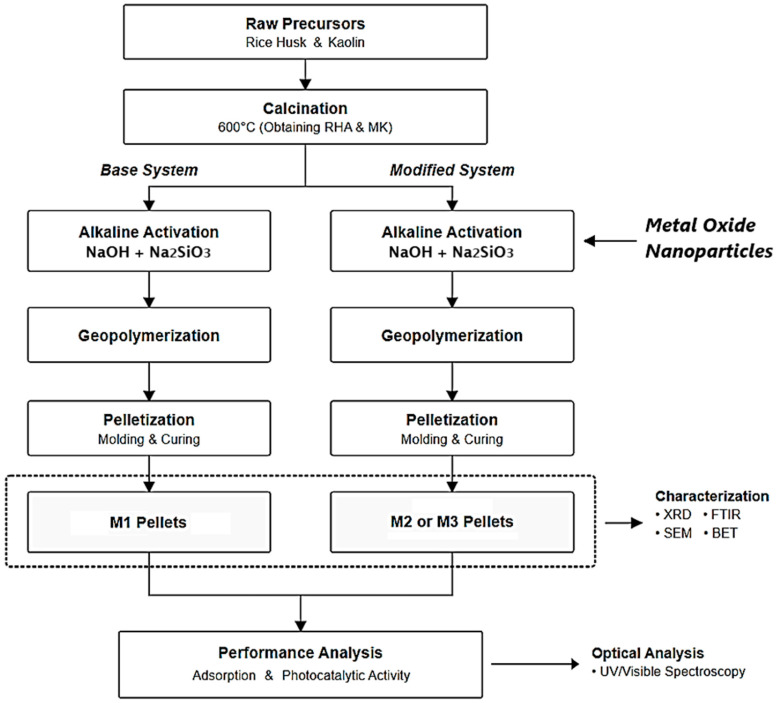
Schematic overview of experimental methodology.

Figure 1 provides a schematic overview of the methodological approach used in this study.

### 2.1. Materials

The following reagents of analytical grade were supplied by Sigma-Aldrich (St. Louis, MO, USA) and used as received without further purification: titanium (IV) isopropoxide (Ti(OC_3_H_7_)_4_, 98.0%), zinc acetate ((CH_3_CO_2_)_2_Zn, 99.99%), isopropyl alcohol (C_3_H_8_O, ≥99.5%), Iron(III) chloride (FeCl_3_, 97%), hydrochloric acid (HCl, 37.0%), sodium hydroxide (NaOH, ≥85.0%), hydrogen peroxide (H_2_O_2_, 35%), sodium metasilicate nonahydrate (Na_2_O_3_Si·9H_2_O, ≥98.0%), kaolin, sodium dodecyl sulfate (CH_3_(CH_2_)_11_OSO_3_Na, ≥99.0%), poly(ethylene glycol) (H(OCH_2_CH_2_)_n_OH).

### 2.2. Synthesis of M1, M2 and M3 Polymers

Firstly, the rice husks (RH) were ground and then calcined at 600 °C to obtain rice husk ash (RHA). Simultaneously, kaolin was calcined at 650 °C for 3 h to induce dehydroxylation and produce metakaolin (MK). The geopolymer compounds were synthesized following a procedure similar to that described by other authors [51]. For the unmodified geopolymer, designated M1, the alkaline activator solution was prepared by dissolving 0.8 g of NaOH in 4.4 g of sodium silicate solution, followed by the addition of 1.5 mL of distilled water. Subsequently, 5.0 g of a mixture of metakaolin and rice husk ash were added to the activator solution and sonicated until completely homogenized. Subsequently, 0.5 g of sodium dodecyl sulfate and 0.5 mL of hydrogen peroxide were added, and the mixture was then sociated again until a homogeneous paste was obtained. From the resulting paste, cylindrical pellets 1.0 cm long and 0.25 cm in diameter were formed using a syringe with an internal diameter of 2.5 cm and allowed to stand for approximately 1 h. The pellets were then immersed in a polyethylene glycol (PEG) bath at 85 ± 5 °C to promote geopolymerization and crosslinking reactions. Once solidified, they were removed from the PEG bath and cured at 45 °C and 65% relative humidity for 24 h to complete the formation of the geopolymer structure. To prepare the modified geopolymer compounds, designated M2 (Geopolymer/Fe_3_O_4_) and M3 (Geopolymer/ZnTiO_3_/TiO_2_), the same synthesis procedure was followed, incorporating 1.0 g of Fe_3_O_4_ nanoparticles or 1.0 g of ZnTiO_3_/TiO_2_, respectively, into the precursor mixture. According to the formulation shown in Table 1, this corresponds to the partial substitution of metakaolin in the solid fraction, resulting in a nominal oxide loading design to evaluate the effect of each functional nanophase on the geopolymer performance. The incorporation of the metallic oxides into the metakaolin matrix was achieved by mechanical mixing prior to geopolymer synthesis [52].

ZnTiO_3_/TiO_2_ nanoparticles were synthesized using a modified sol–gel method based on previously reported procedures [53]. Titanium (IV) isopropoxide (Ti(OC_3_H_7_)_4_) was used as the titanium precursor, while zinc acetate dihydrate (Zn(CH_3_COO)_2_·2H_2_O) served as the zinc source. Initially, titanium isopropoxide was dissolved in isopropanol under continuous magnetic stirring. Subsequently, an aqueous solution containing zinc acetate was slowly added to the reaction mixture to promote controlled hydrolysis and condensation reactions. The molar ratio of TiO_2_:ZnO was fixed at 3:1, which favors the formation of ZnTiO_3_/TiO_2_ mixed oxide phases. The reaction mixture was maintained under stirring at room temperature until the formation of a homogeneous gel. The obtained gel was dried at 60 °C for 24 h and subsequently calcined at 500 °C for 4 h to obtain crystalline ZnTiO_3_/TiO_2_ nanoparticles. On the other hand, Fe_3_O_4_ nanoparticles were synthesized through a solvothermal method adapted from previously reported procedures [54]. In this method, iron precursors were dissolved in a polyol-based solvent system composed of diethylene glycol and ethylene glycol, which acts both as solvent and reducing agent. The reaction mixture was transferred to a sealed autoclave and heated under solvothermal conditions, promoting the nucleation and growth of magnetite (Fe_3_O_4_) nanoparticles. After the reaction, the obtained particles were separated, washed several times with distilled water and ethanol to remove residual reactants, and finally dried before their incorporation into the geopolymeric matrix.

After the curing process, the geopolymers M1, M2 and M3 were pelletized and characterized, and then evaluated in terms of their adsorption and photodegradation capacity against methylene blue (MB) and methyl orange (MO) dyes in aqueous solutions.

### 2.3. Characterization

A comprehensive characterization of the synthesized materials was carried out using several analytical techniques. The crystalline structure of the samples was examined by X-ray diffraction (XRD) using a Bruker AXS D8-Discover diffractometer (Bruker AXS, Karlsruhe, Germany) equipped with a Cu Kα radiation source (λ = 1.5406 Å). Diffraction patterns were recorded over a 2θ range from 5° to 90°. The elemental composition of the samples was determined by X-ray fluorescence (XRF) employing a portable Bruker S1 Turbo SDR spectrometer (Bruker Handheld LLC, Kennewick, WA, USA). Measurements were conducted using the Mining Light Elements analytical mode provided by the instrument. Specific surface area (SSA) measurements were obtained through nitrogen adsorption experiments conducted at −196 °C. The analyses were performed using a ChemiSorb 2720 analyzer (Micromeritics, Norcross, GA, USA) with a gas mixture composed of 30% nitrogen (N_2_) balanced with helium (He). The SSA values were calculated using the Brunauer–Emmett–Teller (BET) isotherm method [55], applying the single-point approximation through the Chemisoft TPx software (version 1.03; Micromeritics, Norcross, GA, USA). Surface morphology and composition were analyzed by scanning electron microscopy (SEM) and energy-dispersive X-ray (EDX), using a JEOL JSM-6400 microscope (SEM-EDX) (JEOL, Peabody, MA, USA). The photocatalytic activity of the materials was evaluated under ultraviolet irradiation at a wavelength of 310 nm, using an IPW-UV-610 stainless steel inner sterilizer lamp (IPW Industries Inc., Santa Ana, CA, USA). Residual concentrations of methylene blue (λ = 665 nm) and methyl orange (λ = 464 nm) in aqueous solutions were quantified by UV–Vis spectrophotometry [56], using a Jenway 7350 spectrophotometer (Cole-Parmer, Staffordshire, UK). The point of zero charge (pH_PZC_) of all samples was determined at room temperature (20 ± 2 °C) using the pH drift method. In each experiment, 0.1 g of pelletized materials were added to 25 mL of a 0.1 M NaCl solution contained in a 50 mL centrifuge tube. The initial pH (pH_i_) of the suspensions was adjusted to values between 3 and 10 using 0.1 M HCl or NaOH solutions. The samples were then agitated at 250 rpm for 24 h, after which the final pH (pH_f_) of the supernatant was measured. The pH_PZC_ was determined from plots of ΔpH (ΔpH = pH_f_ − pH_i_) versus pH_i_, corresponding to the condition ΔpH = 0. This procedure was repeated using NaCl solutions with concentrations of 0.01 and 0.05 M. All measurements were performed in triplicate, and the reported pH_PZC_ values correspond to the average of the three independent determinations [57].

### 2.4. Adsorption Studies

Adsorption experiments using methylene blue (MB) and methyl orange (MO) dyes in aqueous media were performed to investigate the influence of solution pH, initial dye concentration, temperature, and adsorbent–adsorbate contact time. The experimental data obtained were analyzed using adsorption isotherm and kinetic models through nonlinear least-squares regression fitting [58]. All experiments were conducted in batch mode at room temperature, following procedures previously reported in the literature.

The solution pH was adjusted to 7.0 ± 0.2 using 0.1 M sodium hydroxide (NaOH) or hydrochloric acid (HCl) solutions. In all adsorption assays, a fixed adsorbent dosage of 200 mg L^−1^ of geopolymeric material was employed. To determine the maximum adsorption capacity of MB and MO, the initial concentration of a 500 mL dyes solution was varied between 0.25 and 30 mg L^−1^. The effects of pH, temperature, and contact time on MB adsorption were evaluated using a constant dye concentration of 20 mg L^−1^ in 500 mL of aqueous solution. After adsorption, the residual concentration of MB and MO in solution was determined by UV–Vis spectrophotometry. Quantification was based on a previously established calibration curve (R^2^ = 0.9995) in accordance with the Lambert–Beer law. All experiments were performed in triplicate, and the reported values correspond to the average of the three independent measurements.

The adsorption capacity of the geopolymers was calculated using Equation (1) [59]:

(1) $$q_{e}=\left(C_{0}-C_{e}\right)\times \frac{v}{w}$$

where $C_{0}$ and $C_{e}$ (mg L^−1^) represent the initial and equilibrium concentrations of MB, respectively. The adsorbent mass w is expressed in grams (g), and v denotes the solution volume in liters (L).

To describe adsorption equilibrium, the Langmuir and Freundlich isotherm models were applied. The Langmuir model is given by Equation (2) [60]:

(2) $$\frac{C_{e}}{q_{e}}=\frac{1}{K_{L}q_{max}}+\frac{C_{e}}{q_{max}}$$

where $q_{\max}$ (mg g^−1^) corresponds to the maximum monolayer adsorption capacity, and $K_{L}$ (L mg^−1^) is the Langmuir constant related to adsorption energy. The equilibrium solute concentration is represented by $C_{e}$ (mg L^−1^). In addition, the dimensionless separation factor $R_{L}$, which provides insight into the favorability of the adsorption process, was calculated using Equation (3) [60]:

(3) $$R_{L}=\frac{1}{\left(1+K_{L}C_{0}\right)}$$

Adsorption behavior is classified as linear when $R_{L}=1$, unfavorable when $R_{L}>1$, irreversible when $R_{L}=0$, and favorable when $0<R_{L}<1$.

The Freundlich isotherm model was also employed and is expressed by Equation (4) [60]:

(4) qe=KFCe1n

where $K_{F}$ (L mg^−1^) is the Freundlich constant associated with adsorption capacity, and 1/n describes adsorption intensity. Values of n between 1 and 10 indicate favorable adsorption conditions [61].

Thermodynamic parameters, including Gibbs free energy change (∆G^0^, kJ mol^−1^), enthalpy change (∆H^0^, kJ mol^−1^), and entropy change (∆S^0^, kJ mol^−1^ K^−1^), were determined from adsorption data. The Gibbs free energy change was calculated using Equation (5) [62]:

(5) $$∆G^{0}=-RTlnk_{C}$$

The relationship between ∆G^0^, ∆H^0^, and ∆S^0^ was further analyzed using the Van’t Hoff equation, shown in Equation (6) [62]:

(6) $$\ln k_{C}=\frac{-∆H^{0}}{R}\times \frac{1}{T}+\frac{∆S^{0}}{R}$$

where T is the absolute temperature (K) and R is the universal gas constant (8.314 J mol^−1^ K^−1^). The dimensionless equilibrium constant $k_{C}$ was obtained from the Langmuir constant $k_{L}$ (L mg^−1^) by multiplying it by the molecular weight of the adsorbate ($M_{w}$, g mol^−1^), followed by multiplication with 1000 and 55.5, as shown in Equation (7) [63]:

(7) $$k_{C}=k_{L}\times M_{w}\times 1000\times 55.5$$

Adsorption kinetics were evaluated using several models, including the pseudo-first-order, pseudo-second-order, intraparticle diffusion, external-film diffusion, and internal-pore diffusion models. The pseudo-first-order kinetic model is described by Equation (8) [61]:

(8) $$\ln\left(q_{e}-q_{t}\right)=\ln\left(q_{e}\right)-k_{1}t$$

where $k_{1}$ (min^−1^) is the pseudo-first-order rate constant, and $q_{e}$ and $q_{t}$ (mg g^−1^) represent the amount of dye adsorbed at equilibrium and at time t, respectively.

The pseudo-second-order kinetic model is expressed by Equation (9) [61]:

(9) $$\frac{t}{q_{t}}=\frac{1}{k_{2}q_{e}^{2}}+\frac{1}{q_{e}}t$$

where k_2_ (g mg^−1^ min^−1^) is the pseudo-second-order rate constant.

To further elucidate the adsorption mechanism and identify the rate-controlling steps, the intraparticle diffusion model was applied using Equation (10) [61]:

(10) qt=k3t12+A

where $k_{3}$ (mg g^−1^ min^−1/2^) is the intraparticle diffusion rate constant, and A (mg g^−1^) is a constant related to boundary layer thickness. Larger values of A indicate a greater boundary layer effect. The presence of multiple linear regions in plots of $q_{t}$ versus $t^{1/2}$ suggests that adsorption occurs through multiple diffusion stages.

The internal pore diffusion model was also considered. When adsorption is controlled by particle diffusion, the process is described by Equation (11) [61]:

(11) $$-ln\left(1-{\left(\frac{q_{t}}{q_{e}}\right)}^{2}\right)=\frac{2\pi^{2}D_{p}}{r^{2}}t$$

Alternatively, if the adsorption rate is governed by external-film diffusion, Equation (12) applies [61]:

(12) $$-ln\left(1-\left(\frac{q_{t}}{q_{e}}\right)\right)=\frac{D_{f}C_{s}}{hrC_{z}}t$$

In these equations, $q_{e}$ and $q_{t}$ (mg g^−1^) denote the adsorption capacities at equilibrium and at time t, respectively. The solute concentrations in the liquid and solid phases are represented by $C_{s}$ (mg L^−1^) and $C_{z}$ (mg kg^−1^). The parameter r corresponds to the average particle radius (1 × 10^−7^ m), while h represents the thickness of the liquid film surrounding the particles, assumed to be 10^−6^ m under mild agitation. The diffusion coefficients in the adsorbent and film phases are denoted by $D_{p}$ (m^2^ min^−1^) and $D_{f}$ (m^2^ min^−1^), respectively.

### 2.5. Photodegradation Studies

Photocatalytic degradation tests were conducted under heterogeneous conditions following a protocol adapted from our previous work [64]. All experiments were performed in batch reactors equipped with ultraviolet lamps emitting radiation at a wavelength of 310 nm. For each assay, the geopolymeric materials were dispersed in aqueous media at a fixed concentration of 0.2 g L^−1^.

The reaction system consisted of 500 mL of aqueous solution adjusted to pH 7, containing methylene blue (MB) at an initial concentration of 20 mg L^−1^ [64]. During irradiation, aliquots were collected at predetermined time intervals to monitor the degradation of the dye.

The photodegradation kinetics were analyzed using the Langmuir–Hinshelwood model [65], which is expressed by Equation (13) [66]:

(13) $$ln\frac{C_{o}}{C_{t}}=kKt=k_{app}t$$

where k (min^−1^) represents the intrinsic reaction rate constant and K corresponds to the adsorption equilibrium constant of the dye on the geopolymeric surface. The dye concentration at the initial time is denoted as $C_{0}$ (mg L^−1^), while $C_{t}$ (mg L^−1^) refers to the concentration at a given irradiation time t (min). The apparent rate constant $k_{\mathrm{app}}$ (min^−1^) was obtained from the slope of the linear plot of $ln(C_{0}/C_{t})$ versus time, with the intercept fixed at zero.

### 2.6. Total Removal Efficiency and Reusability Test

To evaluate the reusability of the synthesized geopolymeric materials in photocatalytic applications, recycling experiments were performed under combined adsorption–photodegradation conditions. The reusability assessment consisted of five consecutive treatment cycles carried out using the same experimental configuration described above. At the end of each cycle, the reaction suspensions were allowed to settle for at least 1 h, after which the supernatant liquid was carefully decanted. The recovered solid materials were subsequently washed using a methanol solution containing 6% (v/v) acetic acid, which acted as the eluent. After the washing step, the geopolymeric compounds were dried and reused in the subsequent cycle. In each reuse cycle, a fresh MB solution with an initial concentration of 20 mg L^−1^ was treated using an adsorbent dosage of 200 mg L^−1^. The experimental procedure adopted for the reusability tests was based on previously reported studies [64].

## 3. Results

### 3.1. Synthesis of the Geopolymers

Three different rice husk ash (RHA)/metakaolin mass ratios (25:75, 50:50, and 75:25) were evaluated to identify the optimal formulation for geopolymer synthesis. The yield reported in this study refers to the residual mass integrity of the geopolymeric pellets after undergoing resistance tests, which consisted of immersing the pellets in aqueous solutions and subjecting them to rotary agitation for 24 h. In this context, a higher yield indicates greater structural stability, as it corresponds to a lower degree of pellet disintegration during the test. The results indicate that the 25:75 ratio exhibited the highest yield, reaching 95.37%, whereas the 50:50 and 75:25 formulations showed significantly lower values of 78.31% and 72.61%, respectively.

The superior performance of the 25:75 formulation can be attributed to a more favorable chemical interaction between the precursors. Metakaolin (MK), characterized by its high reactivity and alumina content, effectively complements the amorphous silica-rich rice husk ash, promoting the development of a continuous aluminosilicate geopolymeric network. This balanced Si/Al contribution facilitates polycondensation reactions, resulting in improved structural integrity, higher yield, and enhanced porosity. In contrast, formulations with higher rice husk ash content exhibited reduced efficiency, likely due to insufficient reactive alumina, which limits the extent of geopolymerization and leads to incomplete matrix formation. Consequently, the 25:75 RHA/MK ratio was selected as the optimal composition for subsequent modifications and performance evaluations.

**Figure 2:**
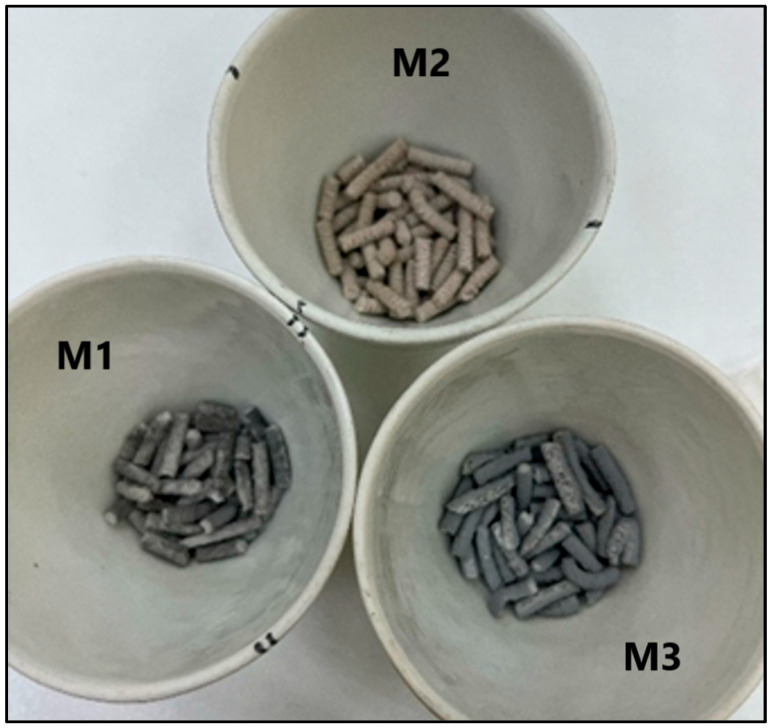
Pelletized geopolymers M1, M2 and M3.

Based on the 25:75 RHA/MK formulation, three geopolymer systems, designated M1, M2, and M3, were considered, differing only in the type of additive incorporated. Figure 2 shows the materials synthesized after the pelletizing process.

All samples were prepared using identical amounts of sodium hydroxide (NaOH), sodium silicate (Na_2_SiO_3_), sodium lauryl ether sulfate (CH_3_(CH_2_)_11_OSO_3_Na) as surfactant, hydrogen peroxide (H_2_O_2_) as pore-forming agent, metakaolin (MK), and calcined rice husk ash (RHA). As shown in Table 1, sample M1 corresponds to the unmodified geopolymer matrix, serving as the reference material. Sample M2 was functionalized with iron oxide (Fe_3_O_4_), while sample M3 was modified with zinc titanate/anatase (ZnTiO_3_/TiO_2_).

These compositional variations enable systematic evaluation of the influence of iron- and titanium-based modifiers on the structural characteristics and environmental performance of the resulting geopolymeric materials.

### 3.2. XRF and XRD Analysis

Table 2 summarizes the chemical composition of rice husk ash (RHA) and the synthesized geopolymer samples (M1, M2, and M3) as determined by X-ray fluorescence (XRF) analysis. These results confirm the successful incorporation of the modifying nanophases into the geopolymer matrix. In the Fe_3_O_4_-modified geopolymer (M2), the Fe_2_O_3_ content determined by XRF (8.22 wt%) corresponds to approximately 7.9 wt% of Fe_3_O_4_-equivalent phase considering the stoichiometric relationship between Fe_2_O_3_ and Fe_3_O_4_. In the ZnTiO_3_/TiO_2_-modified geopolymer (M3), the XRF analysis reveals TiO_2_ and ZnO contents of 6.13 wt% and 1.99 wt%, respectively. These values are consistent with the nominal TiO_2_:ZnO ratio of approximately 3:1 used during nanoparticle synthesis. The total oxide fraction associated with the semiconductor phase therefore corresponds to approximately 8.1 wt% of ZnTiO_3_/TiO_2_-related material in the geopolymer. Overall, these results indicate that the loading of functional nanoparticles in the hybrid geopolymers is on the order of ~8 wt%.

**Figure 3:**
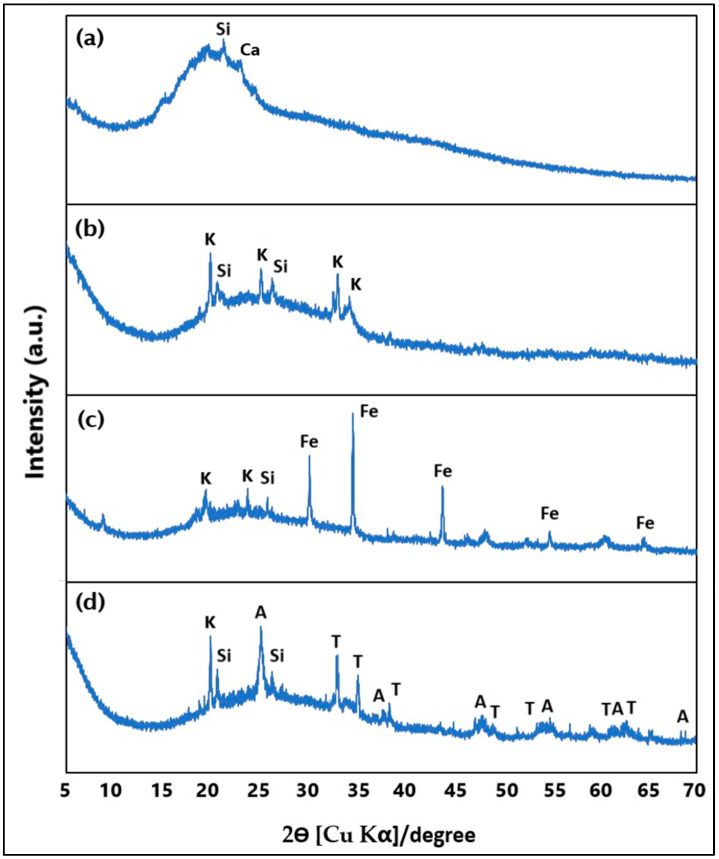
X-ray diffraction pattern of (**a**) Rice husk ash, (**b**) Geopolymer, (**c**) Fe_3_O_4_-modified geopolymer and (**d**) ZnTiO_3_/TiO_2_-modified geopolymer. Si: Silicon Oxide, Ca: Calcium carbonate, K: Kaolin, Fe: Iron Oxide, T: Zinc Titanate, A: Anatase.

Furthermore, Figure 3 presents the X-ray diffraction patterns of (a) rice husk ash (RHA), (b) geopolymer (M1), (c) Fe_3_O_4_-modified geopolymer (M2), and (d) ZnTiO_3_/TiO_2_-modified geopolymer (M3). The diffraction pattern of RHA is characterized by a broad diffuse band centered between approximately 15° and 30° (2θ), along with low-intensity reflections attributed to silicon and calcium-containing phases. The XRD pattern of the geopolymer sample (M1) displays a broad background with superimposed diffraction peaks corresponding to silicon- and aluminosilicate-related phases, as well as minor crystalline contributions probably associated with potassium- and calcium-containing compounds. In the Fe_3_O_4_-modified geopolymer (M2), additional well-defined diffraction peaks assigned to iron oxide phases are observed, while the overall diffractogram retains the broad background typical of the geopolymer matrix. The average crystallite size of Fe_3_O_4_ was estimated using the Scherrer equation and found to be approximately 38.46 nm, indicating the formation of nanocrystalline magnetite particles. These observations are consistent with previous reports for similar systems [54,67]. For the ZnTiO_3_/TiO_2_-modified geopolymer (M3), the diffraction pattern shows characteristic reflections corresponding to ZnTiO_3_ and TiO_2_ phases, together with the background signal associated with the geopolymer matrix. The average crystallite size of the ZnTiO_3_/TiO_2_ particles was calculated to be 34.46 ± 5.11 nm using the Scherrer equation. These observations are consistent with previous reports for similar systems [68]. In all samples, the main diffraction features appear within the 5–70° (2θ) range, and the presence of distinct crystalline phases varies according to the incorporated modifiers.

### 3.3. FTIR Analysis

**Figure 4:**
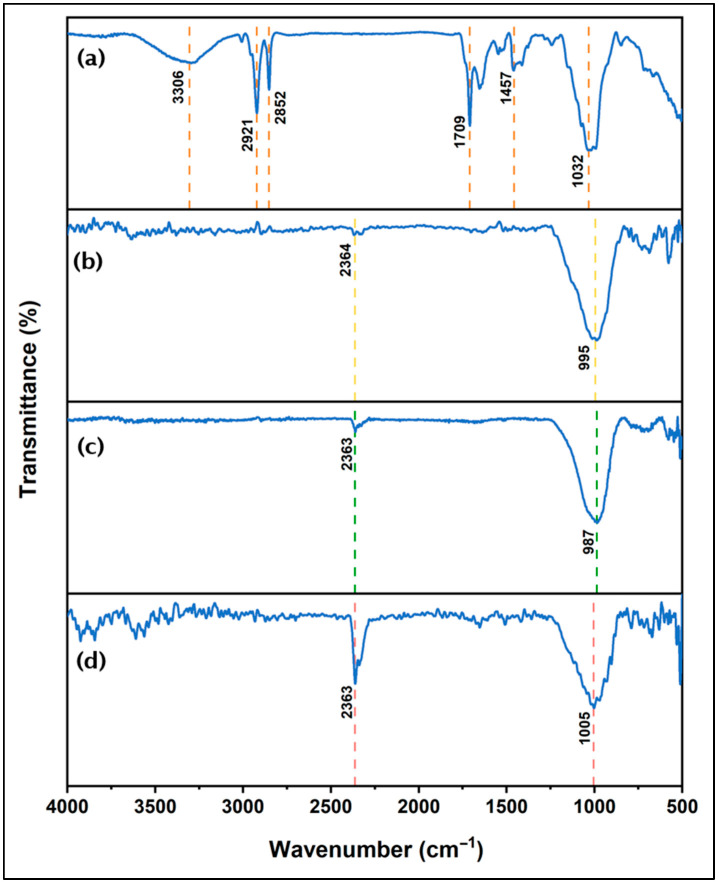
Infrared spectrum of (**a**) Husk rice ash, (**b**) Geopolymer, (**c**) Fe_3_O_4_-modified geopolymer and (**d**) ZnTiO_3_/TiO_2_ modified geopolymer.

Figure 4 shows the FTIR spectra of (a) rice husk ash (RHA), (b) geopolymer (M1), (c) Fe_3_O_4_-modified geopolymer (M2), and (d) ZnTiO_3_/TiO_2_-modified geopolymer (M3) recorded in the range 500–4000 cm^−1^.

The FTIR spectrum of RHA (Figure 4a) exhibits several absorption bands distributed across the entire spectral range. In the high-wavenumber region, a broad band is observed between approximately 3250 and 3500 cm^−1^. Additional absorption bands appear at 2921 cm^−1^ and 2852 cm^−1^. A distinct band is detected at 1709 cm^−1^, along with another band at 1457 cm^−1^. The most intense absorption band is located at 1032 cm^−1^, while lower-intensity bands are observed below 600 cm^−1^. The FTIR spectrum of the geopolymer sample (M1, Figure 4b) shows a dominant absorption band centered at approximately 995 cm^−1^. A weak band is observed around 2364 cm^−1^, while the intensity of bands in the high-wavenumber region is significantly reduced compared to RHA. For the Fe_3_O_4_-modified geopolymer (M2, Figure 4c), the spectrum is characterized by a main absorption band at approximately 987 cm^−1^ and a weak band around 2363 cm^−1^. The overall spectral profile is similar to that of the unmodified geopolymer. The ZnTiO_3_/TiO_2_-modified geopolymer (M3, Figure 4d) exhibits its main absorption band at approximately 1005 cm^−1^, accompanied by a weak band near 2363 cm^−1^. As observed for M1 and M2, no additional intense bands appear in the high-wavenumber region, and the spectra are dominated by the absorption features associated with the geopolymer matrix.

### 3.4. SEM and SSA Analysis

**Figure 5:**
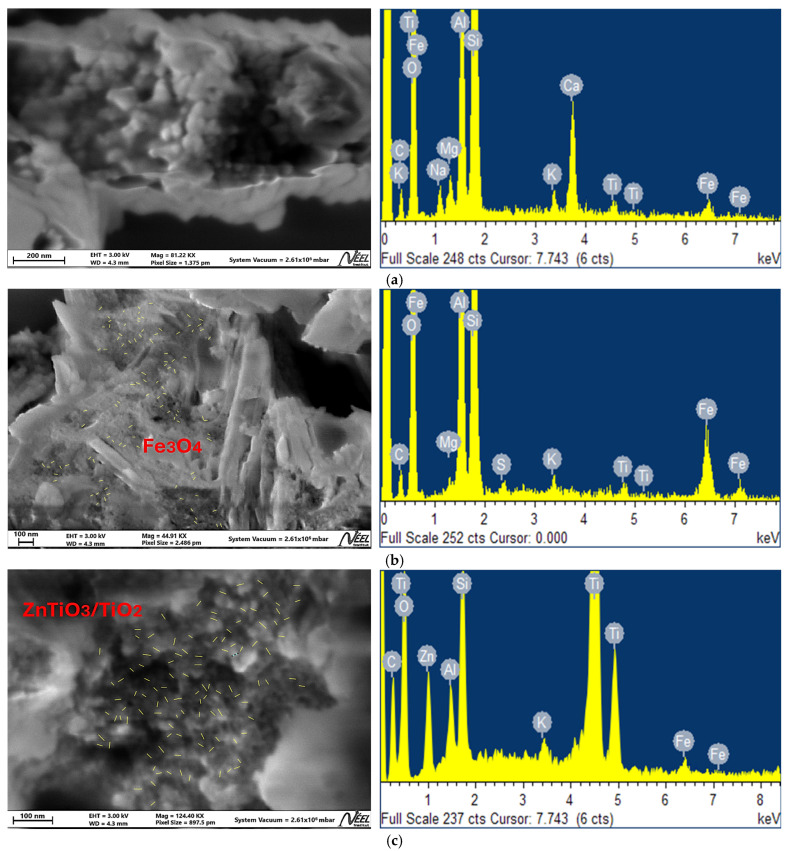
SEM images and EDX spectra of (**a**) Geopolymer, (**b**) Fe_3_O_4_-modified geopolymer and (**c**) ZnTiO_3_/TiO_2_ modified geopolymer. The yellow lines on the micrographs highlight some Fe_3_O_4_ and ZnTiO_3_/TiO_2_ nanoparticles.

The SEM image of the unmodified geopolymer M1 (Figure 5a) reveals a heterogeneous and relatively compact morphology, typical of aluminosilicate geopolymeric matrices. Irregular agglomerates with rough surfaces are observed, together with dense regions and limited porosity unevenly distributed across the surface. In contrast, the micrograph of sample M2 (Figure 5b), corresponding to the geopolymer modified with Fe_3_O_4_ nanoparticles, exhibits a more complex surface morphology. The Fe_3_O_4_-modified geopolymer (M2) (Figure 5b) presents the presence of nanoparticulate domains distributed within the aluminosilicate matrix. The Fe_3_O_4_ particles exhibit a nanometric size with an average diameter of approximately 27 nm. These particles appear highly agglomerated, forming compact clusters embedded in the geopolymeric framework. The surface morphology is heterogeneous, with irregular aggregates and regions of increased surface roughness. The ZnTiO_3_/TiO_2_-modified geopolymer (M3) (Figure 5c) shows a distinct morphological profile. The semiconductor nanoparticles display a smaller average particle size of approximately 24 nm. Compared to M2, the nanoparticles in M3 appear less agglomerated and more uniformly distributed throughout the matrix. The average particle size was estimated from SEM micrographs using ImageJ2 software (version 2.17.0; National Institute of General Medical Sciences, Bethesda, MD, USA) through measurements of multiple particles in representative regions of the images [69]. The nanoparticle-rich regions corresponding to the Fe_3_O_4_ and ZnTiO_3_/TiO_2_ phases are directly indicated in the SEM micrographs to facilitate their identification within the geopolymer matrix. These regions exhibit a finer granular texture and a more homogeneous arrangement of aggregates.

The elemental composition of samples M1, M2, and M3 was analyzed by EDX (Figure 5). The unmodified geopolymer (M1) shows predominant O, Si, and Al signals, confirming its aluminosilicate matrix, along with minor Na, Mg, K, Ca, and Fe from the precursors. In M2, a marked increase in Fe intensity confirms the incorporation of Fe_3_O_4_ nanoparticles. In M3, distinct Zn and Ti peaks are observed, with a significantly higher Ti signal compared to M1 and M2, indicating successful incorporation of the ZnTiO_3_/TiO_2_ phase. In all modified samples, the persistence of Si, Al, and O signals confirms preservation of the geopolymeric framework. Table 3 shows the percentage composition (wt%) of the elements present in the M1, M2 and M3 geopolymers. These values corroborate the results shown in the EDX spectra of Figure 5.

Regarding specific surface area (SSA), this varies depending on the surface modification and the sample shape. Table 4 shows higher SSA values for the modified powder geopolymers and a notable reduction after pelletizing. The nitrogen adsorption–desorption curves obtained during the analysis based on the BET isotherm method are provided in the Supplementary Materials (Figure S1), showing the adsorption behavior of the synthesized materials.

In this study, the pelletized configuration was selected to facilitate material handling, recovery after treatment, and repeated reuse cycles, which are essential aspects for potential practical applications of heterogeneous adsorbents and photocatalysts.

### 3.5. Adsorption Capacity

#### 3.5.1. Effect of the pH of the Solution

**Figure 6:**
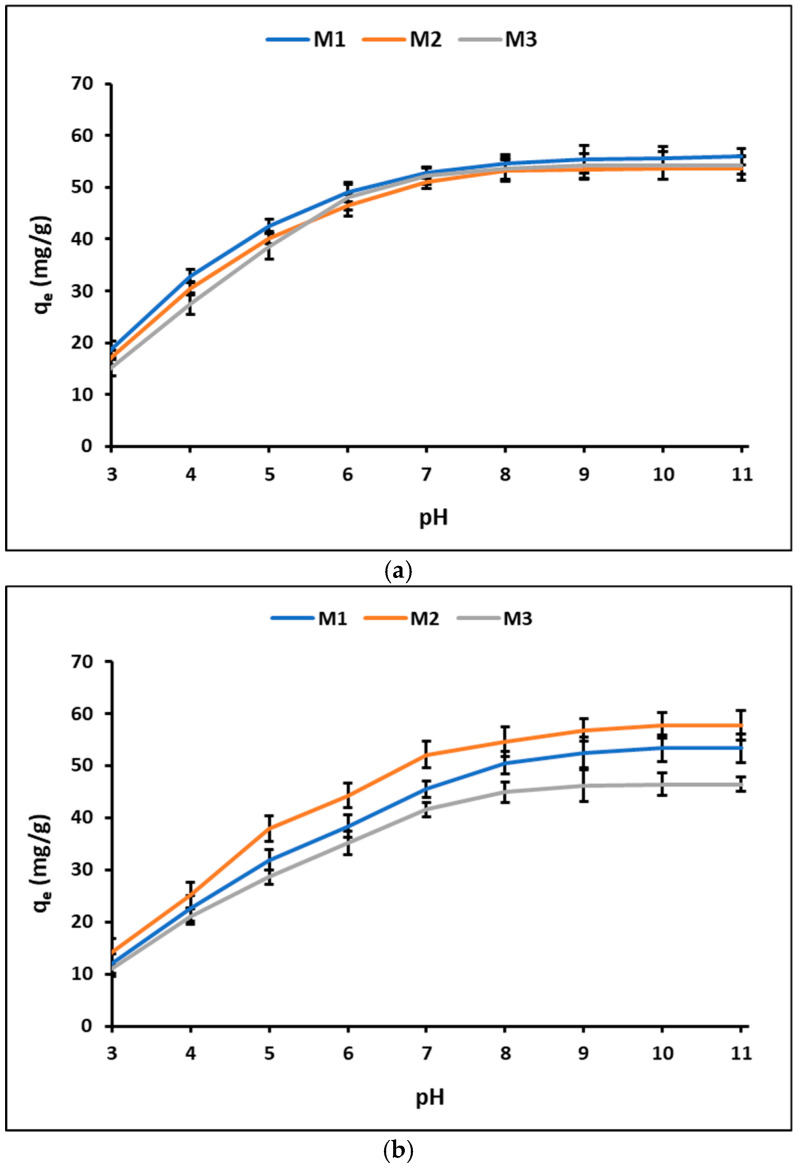
Effect of pH on the adsorption of (**a**) Methyl Blue (MB) and (**b**) Methyl Orange (MO) dyes on M1, M2 and M3 geopolymers.

The pH_PZC_ values determined for the geopolymer samples were 6.7 for the unmodified geopolymer (M1), 6.9 for the Fe_3_O_4_ modified geopolymer (M2), and 7.2 for the ZnTiO_3_/TiO_2_ modified geopolymer (M3). These values allow us to establish the surface charge behavior of the materials and serve as a reference for analyzing the effect of the solution pH on adsorption capacity. In this context, Figure 6 shows the influence of the solution pH on the adsorption capacity (q_e_) of the geopolymer granules (M1, M2, and M3) for methylene blue (MB) and methyl orange (MO).

For methylene blue (Figure 6a), the adsorption capacity of all samples increases progressively as the pH rises from acidic to alkaline conditions. At low pH values, relatively low adsorption capacities are observed. As the pH increases, q_e_ rises steadily, showing a marked increase between pH 4 and 7. The adsorption capacity reaches a maximum at approximately pH 8–9, after which the curves tend to stabilize, exhibiting only minor variations at higher pH values. Across the entire pH range, the three geopolymeric samples display similar adsorption trends. In the case of methyl orange (Figure 6b), a comparable pH-dependent behavior is observed. The adsorption capacity increases gradually as the pH increases from acidic values. A pronounced rise in q_e_ occurs between pH 4 and 7, followed by a stabilization of adsorption capacity at near-neutral to slightly alkaline pH values. Maximum adsorption is achieved at approximately pH 7–8, with no significant changes observed at higher pH levels. As with methylene blue, the adsorption trends for M1, M2, and M3 remain closely aligned throughout the studied pH range. Based on these results, pH 7 was selected for further adsorption and photodegradation studies.

#### 3.5.2. Effect of Initial Dye Concentration

Table 5 summarizes the isotherm parameters obtained for methylene blue (MB) and methyl orange (MO) adsorption onto the geopolymeric pellets. Tables S1–S6 show the equilibrium adsorption data of the dyes on geopolymers. The experimental equilibrium data were fitted to both Langmuir and Freundlich models. Figures S1–S6 show the linear fit of the Langmuir and Freundlich isotherm models for the adsorption of the dyes on geopolymers. For all samples, the Langmuir model exhibits higher correlation coefficients and lower error values compared to the Freundlich model. These results indicate that the adsorption behavior of both dyes is more accurately described by the Langmuir isotherm under the studied conditions.

#### 3.5.3. Effect of Reaction Temperature

Table 6 presents the thermodynamic parameters associated with the adsorption of methyl blue (MB) and methyl orange (MO) onto the geopolymers at three different temperatures (293.15, 298.15, and 303.15 K). For all systems, negative values of Gibbs free energy change (∆G°) are obtained at the evaluated temperatures, indicating that the adsorption process occurs spontaneously across the studied temperature range. The ∆G° values become slightly more negative as temperature increases for both dyes and all geopolymeric samples. The enthalpy change (∆H°) values determined for MB and MO adsorption are negative for all compounds. For MB, ∆H° values range between −9.66 and −13.96 kJ mol^−1^, while for MO they vary between −10.52 and −14.40 kJ mol^−1^. The entropy change (∆S°) values are positive and remain within a narrow range, between 0.18 and 0.19 kJ mol^−1^ K^−1^, regardless of the dye or geopolymer composition.

#### 3.5.4. Effect of Contact Time

In this study, the adsorption kinetics of methylene blue (MB) and methyl orange (MO) onto the geopolymeric materials were evaluated using the Lagergren pseudo-first-order and Ho pseudo-second-order kinetic models. Additional kinetic descriptions were obtained using the intraparticle diffusion model. The experimental kinetic data and the corresponding model parameters are summarized in Table 7. According to the values reported in this table, the pseudo-second-order model yields higher correlation coefficients and lower χ^2^ values compared to the pseudo-first-order model for both dyes and all samples, indicating a chemisorption process [70].

**Figure 7:**
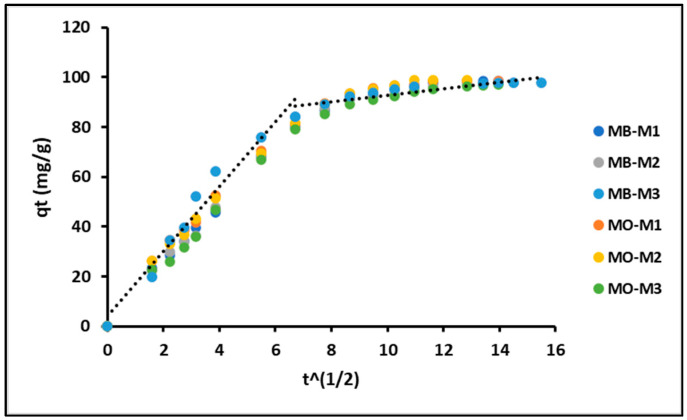
Intra-particle diffusion plots for MB and MO adsorption on M1, M2 and M3 geopolymers.

The intraparticle diffusion model was employed to elucidate the adsorption rate, considering the transfer rate of MB and MO molecules from the aqueous solution to the adsorption sites on the geopolymeric compounds. Figure 7 exhibits the evolution of the q_t_ (mg g^−1^) curves in relation to the square root of time (t^1/2^) for the geopolymers. The figure demonstrates that the adsorption process can be divided into two distinct linear regions. Hence, the MB and MO adsorption process can be described as a combination of film diffusion initially, followed by a subsequent particle diffusion process [71].

### 3.6. Photocatalytic Activity

**Figure 8:**
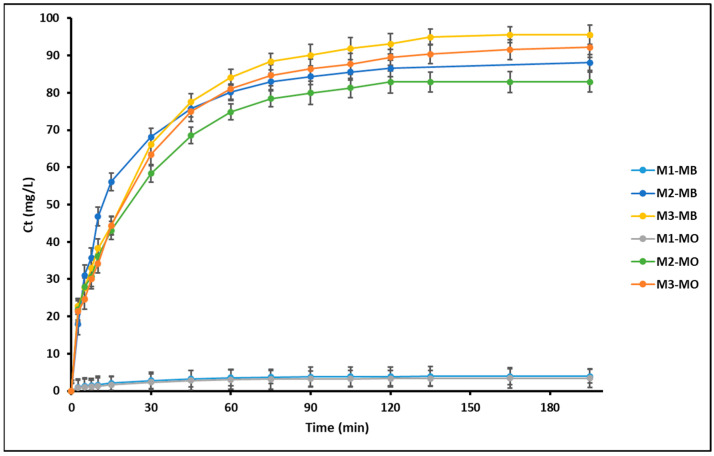
Photocatalytic dye degradation by M1, M2 and M3 geopolymers.

Figure 8 shows the photodegradation profiles of methylene blue and methyl orang in aqueous solution under ultraviolet irradiation in the presence of the synthesized geopolymeric compounds. The evolution of dye concentration as a function of irradiation time was monitored for the unmodified geopolymer (M1) and the Fe_3_O_4_- and ZnTiO_3_/TiO_2_-modified geopolymers (M2 and M3). For both dyes, a rapid decrease in concentration is observed during the initial stage of irradiation, followed by a slower removal rate as the reaction progresses. In all cases, the concentration profiles tend to approach a plateau after approximately 90 min of UV exposure. The modified geopolymers (M2 and M3) exhibit a markedly higher dye removal compared to the unmodified geopolymer throughout the entire irradiation period.

The photodegradation kinetics of MB and MO were evaluated using the Langmuir–Hinshelwood model. A linear relationship between ln(C_0_/Ct) and irradiation time was obtained for all systems, indicating that the photodegradation process follows apparent pseudo-first-order kinetics under the experimental conditions. The apparent rate constants (k_app_) were determined to be 0.017 min^−1^ for the unmodified geopolymer (M1), 0.029 min^−1^ for the Fe_3_O_4_-modified geopolymer (M2), and 0.032 min^−1^ for the ZnTiO_3_/TiO_2_-modified geopolymer (M3).

### 3.7. Efficiency and Reuse of the Geopolymers

**Figure 9:**
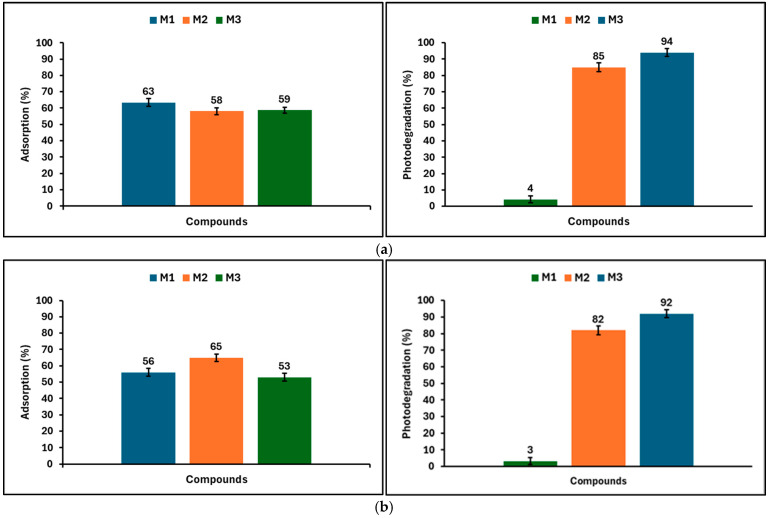
Efficiency of adsorption and photodegradation on the compounds of (**a**) Methylene Blue (MB) and (**b**) Methyl Orange (MO).

Figure 9 summarizes the adsorption and photocatalytic efficiencies of the geopolymeric compounds (M1, M2, and M3) toward methylene blue (MB) and methyl orange (MO). For MB (Figure 9a), the adsorption efficiencies of the geopolymeric materials range between approximately 58% and 63%. The unmodified geopolymer (M1) exhibits the highest adsorption efficiency (63%), followed by the ZnTiO_3_/TiO_2_-modified geopolymer (M3, 59%) and the Fe_3_O_4_-modified geopolymer (M2, 58%). In contrast, significant differences are observed in the photocatalytic degradation efficiencies of MB. The unmodified geopolymer shows a very low photodegradation efficiency (approximately 4%), whereas the Fe_3_O_4_-modified geopolymer achieves a degradation efficiency of about 85%. The ZnTiO_3_/TiO_2_-modified geopolymer exhibits the highest photodegradation efficiency, reaching approximately 94%. For MO (Figure 9b), the adsorption efficiencies range between approximately 53% and 65%. The Fe_3_O_4_-modified geopolymer (M2) shows the highest adsorption efficiency (65%), followed by the unmodified geopolymer (M1, 56%) and the ZnTiO_3_/TiO_2_-modified geopolymer (M3, 53%). Regarding photocatalytic performance, the unmodified geopolymer again exhibits negligible activity, with a photodegradation efficiency of approximately 3%. In contrast, the Fe_3_O_4_-modified geopolymer reaches a degradation efficiency of about 82%, while the ZnTiO_3_/TiO_2_-modified geopolymer shows the highest efficiency, close to 92%.

**Figure 10:**
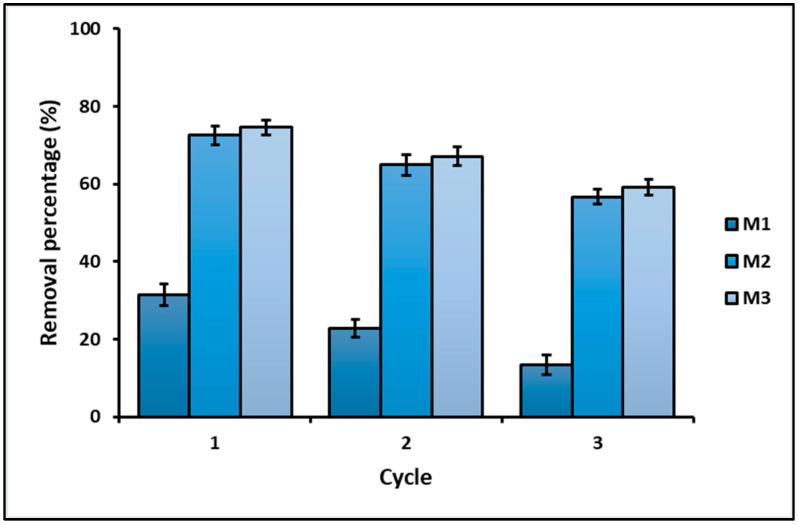
Reusability performance of geopolymeric materials under combined adsorption–photodegradation cycles.

Finally, Figure 10 shows the removal efficiency of the geopolymeric materials (M1, M2, and M3) during three consecutive treatment cycles, where each cycle includes an initial adsorption step followed by photocatalytic degradation under UV irradiation. In the first cycle, the modified geopolymers (M2 and M3) exhibit significantly higher removal efficiencies compared to the unmodified geopolymer (M1). The total removal percentage achieved by M2 and M3 exceeds 70%, whereas M1 shows a markedly lower removal efficiency. As the number of cycles increases, a gradual decrease in total removal efficiency is observed for all samples. During the second cycle, the removal percentages decrease for all geopolymeric materials; however, M2 and M3 maintain substantially higher efficiencies than M1. By the third cycle, although a further reduction is evident, the modified geopolymers continue to demonstrate appreciable removal performance, while the unmodified geopolymer shows the lowest efficiency.

## 4. Discussion

### 4.1. Chemical Composition and Structure

The XRD pattern of rice husk ash (Figure 3a) is dominated by a broad diffuse diffraction band centered between approximately 15° and 30° (2θ), characteristic of an amorphous siliceous structure. This feature indicates the absence of long-range crystalline order and is typically associated with amorphous SiO_2_ derived from biomass combustion. Superimposed on this amorphous background, weak reflections partially match reference patterns from the ICDD database corresponding to silicon (Si), silicon oxide (SiO_2_), aluminum oxide (Al_2_O_3_), iron oxide (Fe_2_O_3_), and calcium carbonate (CaCO_3_). These observations are consistent with the XRF results. Overall, the RH sample consists predominantly of amorphous silica with minor crystalline inclusions, which enhances its reactivity as a precursor for geopolymer synthesis.

The XRD pattern of the unmodified geopolymer (Figure 3b) shows a broad amorphous diffraction band, confirming that the geopolymerization process leads to the formation of a predominantly amorphous aluminosilicate network. This amorphous phase is associated with the sodium–aluminum–silicate–hydrate gel (N–A–S–H), which results from the dissolution and polycondensation of metakaolin and rice husk ash under alkaline activation [72]. In addition to the amorphous background, several weak diffraction peaks can be indexed to residual crystalline phases such as metakaolin, silicon oxide and, to a lesser extent, aluminum oxide, calcium carbonate, and others, according to ICDD database matches. These crystalline phases originate from incomplete dissolution of the raw materials and from secondary phases formed during geopolymer curing. The XRF analysis supports these findings, showing a substantial increase in Al_2_O_3_ and SiO_2_ contents compared to RH, confirming the successful incorporation of metakaolin into the geopolymeric matrix.

The XRD pattern of the Fe_3_O_4_-modified geopolymer (Figure 3c) exhibits distinct and well-defined diffraction peaks corresponding to magnetite (Fe_3_O_4_), confirming the successful incorporation of the iron oxide phase into the geopolymeric matrix. These peaks are indexed to a cubic crystal structure consistent with JCPDS card No. 89-0691. Despite the appearance of these Fe_3_O_4_ reflections, the broad amorphous halo of the geopolymer matrix remains clearly visible, suggesting that the incorporation of Fe_3_O_4_ does not significantly disrupt the N–A–S–H gel structure. The XRF results corroborate the DRX analysis, showing a marked increase in Fe_2_O_3_ content in M2 compared to M1, while SiO_2_ and Al_2_O_3_ contents remain high, preserving the aluminosilicate framework.

The XRD pattern of the ZnTiO_3_/TiO_2_-modified geopolymer (Figure 3d) reveals additional diffraction peaks corresponding to ZnTiO_3_ and TiO_2_ phases. XRD analysis indicates that the semiconductor consists of approximately 51% ZnTiO_3_ and 49% TiO_2_ (A). ZnTiO_3_ exhibits a rhombohedral crystal structure (COD card No. 00-026-1500), while TiO_2_ is identified in its anatase tetragonal phase (COD card No. 96-900-9087). As observed for M2, the amorphous geopolymeric halo is preserved in M3, indicating that the incorporation of the ZnTiO_3_/TiO_2_ semiconductor occurs without destroying the fundamental geopolymeric gel structure. XRF data further support these results, showing significant amounts of TiO_2_ and ZnO, while SiO_2_ and Al_2_O_3_ remain the dominant components of the matrix.

### 4.2. Infrared Spectral Characterization

Figure 4a shows the FTIR spectrum of RHA in the 500–4000 cm^−1^ range. The broad band observed between 3250 and 3500 cm^−1^ is mainly associated with the stretching vibrations of hydroxyl groups (–OH), including silanol (Si–OH) and aluminol (Al–OH) groups, as well as hydrogen-bonded water molecules within the porous structure of the material [73,74,75]. The spectrum indicates that RHA retains certain organic functional groups. The sharp peaks at 2921 cm^−1^ and 2852 cm^−1^ correspond to the asymmetric and symmetric stretching vibrations of aliphatic C–H_2_ groups, respectively [73,76]. In addition, the band observed at approximately 1709 cm^−1^ can be attributed to C=O stretching vibrations associated with carbonyl-containing surface groups. In biomass-derived precursors, these bands are commonly attributed to residual oxygenated functional groups originating from partially decomposed organic species, such as carbonyl or carboxyl groups associated with carbonaceous residues derived from hemicellulose and lignin components [73,75]. The absorption band at approximately 1457 cm^−1^ is associated with C–H deformation or in-plane bending vibrations of methylene groups, resulting from residual biomass structures [76]. The most prominent band at 1032 cm^−1^ corresponds to the asymmetric stretching vibration of Si–O bonds, confirming the siliceous nature of RHA [76,77,78]. Finally, the vibrations observed below 600 cm^−1^ are attributed to O–Si–O bending modes, which tend to become more clearly defined as organic matter is progressively removed [73,77,78]. The FTIR spectra of the geopolymeric samples exhibit very similar profiles, suggesting that the fundamental structure of the aluminosilicate gel is preserved regardless of the additives used. The dominant band attributed to the asymmetric stretching of Si–O–T bonds (T = Si, Al) appear at 995 cm^−1^ for the base geopolymer, shifting to 987 cm^−1^ in the Fe-modified system and to 1005 cm^−1^ in the ZnTiO_3_/TiO_2_-containing composite. In particular, the Si–O stretching vibration in RHA, initially observed at 1032 cm^−1^, shifts toward lower wavenumbers after geopolymerization, consistent with the results reported by Toniolo et al. [79]. This shift toward lower frequencies has been associated with an increase in tetrahedral aluminum content in the gel, confirming the formation of the aluminosilicate network [80]. According to the literature, characteristic Fe–O and M–O (M = Zn, Ti) vibrations are typically located in the 580–590 cm^−1^ range [81,82]. However, in these systems, the low-wavenumber region is dominated by vibrations of the aluminosilicate framework (Si–O–Si and Si–O–Al), which are usually located between 400 and 700 cm^−1^ [83,84,85,86]. This overlap hinders the clear resolution of bands associated with the incorporated metal oxides. In agreement with the XRD results, which confirm the presence of crystalline oxide phases, the absence of well-defined FTIR bands suggests that the additives are dispersed within the geopolymeric matrix without significantly altering the aluminosilicate connectivity. The small bands observed around 2400 cm^−1^ are most likely associated with the infrared absorption of HCO_3_^−^ ions [87,88,89].

### 4.3. Textural Properties

The morphological differences observed by SEM between M1, M2, and M3 demonstrate the influence of nanoparticle incorporation on the geopolymeric microstructure. The relatively dense morphology of M1 is consistent with the formation of a continuous aluminosilicate gel typical of unmodified geopolymers, where porosity mainly arises from gel formation and water loss during the curing process. The SEM micrograph of the Fe_3_O_4_-modified geopolymer (M2) (Figure 5b) reveals the presence of highly agglomerated magnetic nanoparticles embedded within the aluminosilicate matrix. The Fe_3_O_4_ particles exhibit an average size of approximately 27 nm, consistent with nanometric dimensions; however, they tend to form compact clusters, leading to localized dense regions within the composite. This agglomeration is characteristic of magnetic nanoparticles due to dipole–dipole interactions and contributes to increased surface roughness and structural heterogeneity. The formation of these aggregates may partially block accessible pores, affecting the effective surface area and mass transfer properties of the material. In contrast, the SEM image of the ZnTiO_3_/TiO_2_-modified geopolymer (M3) (Figure 5c) shows a more homogeneous distribution of semiconductor nanoparticles within the matrix. The ZnTiO_3_/TiO_2_ particles present a smaller average size of approximately 24 nm and exhibit significantly lower agglomeration compared to the Fe_3_O_4_-modified sample. The finer dispersion and reduced particle clustering result in a more uniform microstructure, promoting increased porosity and improved accessibility of active sites. This more controlled nanoparticle distribution is consistent with the higher specific surface area observed for the modified powders and may contribute to enhanced interfacial interactions and photocatalytic performance.

The EDX results confirm the successful incorporation of magnetic and semiconductor nanophases within the geopolymeric matrix. In M1, the predominance of Si, Al, and O confirms the formation of the aluminosilicate framework characteristic of N–A–S–H gel structures. The presence of Na and K is consistent with alkaline activation and residual alkali species. For the Fe_3_O_4_-modified geopolymer (M2), the spectrum exhibits a clear increase in Fe signal intensity compared to M1, confirming the incorporation of iron oxide nanoparticles within the geopolymeric structure. In the ZnTiO_3_/TiO_2_-modified geopolymer (M3), additional peaks corresponding to Zn and Ti are clearly observed. The relative intensity of Ti is significantly higher compared to M1 and M2, confirming successful incorporation of the semiconductor phase. The signals corresponding to Si, Al, and O remain present in M2 and M3, indicating preservation of the aluminosilicate matrix.

The specific surface area (SSA) results reveal a pronounced effect of both chemical modification and physical shaping. The increased BET surface area observed in the modified geopolymers suggests the formation of a more accessible surface structure after nanoparticle incorporation. A larger specific surface area generally provides a greater number of active sites for adsorption and photocatalytic reactions. In heterogeneous photocatalysis, surface area plays a crucial role because it determines the availability of catalytic sites for dye adsorption and subsequent photodegradation. Therefore, the larger surface areas observed in the modified materials may contribute to the higher removal efficiencies obtained for methylene blue and methyl orange. On the other hand, the pelletizing process leads to a substantial reduction in SSA in all samples, particularly in the modified geopolymers. This decrease can be attributed to particle agglomeration, pore collapse, or partial blockage of accessible pores during the shaping and consolidation process. Despite this reduction, pelletized materials offer greater mechanical stability and ease of handling, which is advantageous for practical applications involving repeated use and recovery.

### 4.4. Evaluation of Dye Adsorption

#### 4.4.1. pH-Dependent Adsorption Behavior

The pH_PZC_ values obtained for all geopolymeric materials are close to neutral pH, indicating that their surface charge transitions from positive to negative near environmental pH conditions. The slight increase in pH_PZC_ observed for the Fe_3_O_4_- and ZnTiO_3_/TiO_2_-modified geopolymers suggests that metal oxide incorporation alters the surface chemistry of the geopolymer matrix, likely through the introduction of additional surface hydroxyl groups associated with iron, zinc, and titanium species. The combination of near-neutral pH_PZC_ values and increased surface area in the modified geopolymer powders suggests a balance between surface charge characteristics and textural properties. For pelletized systems, although the accessible surface area decreases, the pH_PZC_ values remain close to neutrality, indicating that surface reactivity is maintained, supporting their suitability for adsorption and photocatalytic applications under environmentally relevant conditions.

The observed influence of pH on dye adsorption is closely related to the surface charge properties of the geopolymeric materials and the ionic nature of the adsorbates. In addition, the acid–base properties of the dyes also play an important role. Methylene blue and methyl orange present pKa values of approximately 3.8 [90] and 3.7 [91], respectively, indicating that at pH values above these pKa values the dyes predominantly exist in their ionic forms, enhancing their interaction with charged adsorption sites on the geopolymer surface.

As determined by the point of zero charge (pH_PZC_), the surface of the geopolymeric compounds transitions from positively charged at acidic pH values to negatively charged at pH values close to and above neutrality. Methylene blue is a cationic dye, and its adsorption is strongly favored at pH values higher than the pH_PZC_ of the materials. Under acidic conditions, protonation of surface functional groups reduces the electrostatic attraction between the adsorbent and the positively charged dye molecules, resulting in lower adsorption capacities. As the pH increases, surface deprotonation leads to a net negative surface charge, enhancing electrostatic interactions with methylene blue cations. This mechanism explains the continuous increase in adsorption capacity and the attainment of maximum removal at pH values around 8–9. Methyl orange, on the other hand, is an anionic dye, and its adsorption behavior involves a more complex interaction mechanism. Although electrostatic repulsion would be expected at higher pH values due to the negatively charged surface, the experimental results show increasing adsorption up to pH values around 7–8. This indicates that non-electrostatic interactions, including hydrogen bonding, surface complexation with metal oxide sites, π–π interactions between aromatic dye molecules and the aluminosilicate framework, and van der Waals forces, also contribute significantly to methyl orange adsorption. Once these interactions dominate, the adsorption capacity stabilizes, leading to the observed plateau at near-neutral pH values.

The slight differences observed among M1, M2, and M3 can be attributed to modifications in surface chemistry induced by the incorporation of Fe_3_O_4_ and ZnTiO_3_/TiO_2_ nanoparticles, which introduce additional metal–oxygen functional groups and alter the distribution of surface hydroxyl sites. These changes slightly modify the surface charge distribution and the availability of adsorption sites, leading to differences in electrostatic interactions and adsorption affinity toward the dye molecules. The fact that both dyes reach maximum adsorption under similar pH conditions close to neutrality is advantageous from an application perspective, as it allows efficient removal of both cationic and anionic dyes without the need for extensive pH adjustment. This behavior highlights the versatility of the synthesized geopolymeric materials for wastewater treatment applications involving chemically diverse contaminants.

#### 4.4.2. Concentration-Dependent Adsorption Behavior

The adsorption isotherm parameters summarized in Table 5 provide relevant insight into the adsorption mechanisms of methylene blue (MB) and methyl orange (MO) on the geopolymeric pellets. For both dyes and all samples, the Langmuir model yields higher correlation coefficients (R^2^ ≈ 0.97–1.00) and lower χ^2^ values compared to the Freundlich model, indicating that the adsorption process is better described by a Langmuir-type behavior. This suggests that adsorption occurs predominantly on energetically uniform sites, leading to monolayer coverage of the adsorbate on the geopolymer surface.

The Langmuir maximum adsorption capacities (q_max_) reveal clear differences in adsorption affinity depending on both the dye and the geopolymer modification. For methylene blue, the unmodified geopolymer (M1) exhibits the highest adsorption capacity, followed by the ZnTiO_3_/TiO_2_-modified (M3) and Fe_3_O_4_-modified (M2) samples. This trend indicates that, for the cationic dye, the aluminosilicate matrix itself provides highly effective adsorption sites, likely associated with negatively charged Si–O^−^ and Al–O^−^ groups. The slightly lower q_max_ values observed for the modified geopolymers suggest that the incorporation of metal oxides may partially block or alter some of these sites, reducing the availability of electrostatically favorable locations for MB adsorption.

In contrast, the adsorption of methyl orange shows a different trend. The Fe_3_O_4_-modified geopolymer (M2) presents the highest q_max_ value among all samples, significantly exceeding those of M1 and M3. This enhanced adsorption capacity indicates that iron-containing surface sites contribute positively to the uptake of the anionic dye. The presence of Fe species may promote additional adsorption mechanisms such as surface complexation or specific interactions between MO molecules and iron hydroxyl groups, which are less prominent in the unmodified geopolymer. The comparatively lower q_max_ values observed for M3 suggest that the ZnTiO_3_/TiO_2_ modification is less favorable for MO adsorption, possibly due to differences in surface chemistry and interaction strength with anionic species.

The Langmuir affinity constant (K_L_) values are relatively similar across all samples for both dyes, indicating comparable adsorption energies once adsorption sites are available. Furthermore, the dimensionless separation factor (R_L_) values are identical and fall well within the range 0 < R_L_ < 1, confirming that the adsorption process is favorable for both MB and MO on all geopolymeric materials.

Although the Freundlich model provides a less accurate fit overall, the Freundlich constants still offer useful complementary information. The n values obtained for both dyes are consistently greater than unity, confirming favorable adsorption conditions. However, the higher χ^2^ values and lower R^2^ values compared to the Langmuir model reinforce the conclusion that surface heterogeneity plays a secondary role, and that adsorption is dominated by uniform, energetically similar sites.

Overall, the isotherm analysis demonstrates that adsorption on the geopolymeric pellets proceeds mainly via monolayer formation on homogeneous surfaces, with adsorption capacity strongly influenced by the nature of both the adsorbate and the surface modification. While the unmodified geopolymer shows superior performance toward the cationic dye, the incorporation of Fe_3_O_4_ significantly enhances the adsorption of the anionic dye, highlighting the tunability of geopolymeric materials for selective removal of chemically different contaminants.

#### 4.4.3. Temperature-Dependent Adsorption Behavior

The negative ∆G° values obtained for both methylene blue and methyl orange adsorption confirm that the adsorption process is thermodynamically spontaneous for all geopolymeric compounds. The increase in the magnitude of ∆G° with temperature suggests that adsorption remains favorable over the studied temperature range, although the process does not require elevated temperatures to proceed efficiently.

The negative ∆H° values indicate that the adsorption of both dyes is exothermic. The magnitude of the enthalpy change, which falls within the range typically associated with physical adsorption or weak chemical interactions, suggests that the adsorption process is governed by a combination of electrostatic interactions, hydrogen bonding, and surface complexation rather than strong covalent bonding. This observation is consistent with the isotherm and pH-dependent adsorption results discussed previously.

The positive ∆S° values reflect an increase in randomness at the solid–liquid interface during the adsorption process. This behavior can be attributed to the displacement of water molecules from the geopolymer surface as dye molecules are adsorbed, leading to a net increase in disorder in the system. The similar ∆S° values obtained for all samples indicate that surface modification does not significantly alter the overall entropy-driven contribution to the adsorption process.

Taken together, the thermodynamic analysis demonstrates that the adsorption of methylene blue and methyl orange onto the geopolymeric materials is spontaneous, exothermic, and energetically favorable under the experimental conditions, supporting their potential application in dye removal from aqueous solutions.

#### 4.4.4. Time-Dependent Adsorption Behavior

The kinetic parameters presented in Table 7 provide important insights into the adsorption mechanisms of methylene blue and methyl orange on the geopolymeric compounds. For both dyes, the pseudo-second-order model shows excellent agreement with the experimental data, as evidenced by correlation coefficients equal to or very close to unity and lower χ^2^ values compared to the pseudo-first-order model. This behavior indicates that the adsorption rate is more accurately described by the pseudo-second-order model, suggesting that the overall process is controlled by surface reactions involving active adsorption sites rather than solely by mass transfer in the solution.

The equilibrium adsorption capacities (q_e_) predicted by the pseudo-second-order model are consistently higher than those obtained from the pseudo-first-order model and are closer to the experimentally observed values. This trend is observed for both MB and MO across all geopolymeric samples, reinforcing the suitability of the pseudo-second-order model to represent the adsorption kinetics. The relatively similar rate constants (k_2_) among the samples indicate comparable adsorption rates, although slight variations suggest that surface modification influences the accessibility and reactivity of adsorption sites.

On the other hand, the intraparticle diffusion analysis reveals that diffusion contributes to the adsorption process but is not the sole rate-controlling step. The high correlation coefficients obtained for both film diffusion (D_f_) and particle diffusion (D_p_) models indicate that adsorption proceeds through multiple stages. The higher values of the external diffusion constants (k_d1_) compared to the internal diffusion constants (k_d2_) suggest that boundary layer diffusion dominates the early stages of adsorption, followed by intraparticle diffusion as equilibrium is approached. The fact that the intraparticle diffusion plots do not pass through the origin further confirms that diffusion is not the only mechanism governing the adsorption process.

### 4.5. Evaluation of Dye Photodegradation

The photocatalytic performance of the synthesized geopolymeric compounds toward methylene blue (MB) and methyl orange (MO) degradation under ultraviolet irradiation is closely associated with the presence and nature of the incorporated semiconductor phases. Both Fe_3_O_4_ and ZnTiO_3_/TiO_2_ are well-established photocatalysts, widely reported for their high photooxidant capacity and efficient generation of reactive species under UV light [92]. In the present study, the photodegradation kinetics of MB and MO were successfully described using the Langmuir–Hinshelwood model, which showed a linear relationship between ln(C_0_/Ct) and irradiation time, confirming that the degradation process follows a pseudo-first-order reaction mechanism.

The apparent rate constants (k_app_) obtained for the geopolymeric systems indicate clear differences in photocatalytic activity depending on the surface modification. The Fe_3_O_4_- and ZnTiO_3_/TiO_2_-modified geopolymers exhibit significantly higher degradation rates compared to the unmodified geopolymer, highlighting the crucial role of the semiconductor phases in promoting photocatalytic reactions. The observed k_app_ values are consistent with those reported in previous studies for similar hybrid photocatalytic systems [93], confirming the reliability of the synthesized materials.

As illustrated in Figure 8, the photodegradation of both dyes proceeds rapidly during the initial stages of irradiation, with a substantial fraction of MB and MO removed within the first 90 min. After this period, the degradation curves tend to level off, indicating that the reaction approaches a kinetic plateau. This behavior is commonly attributed to a reduction in available dye concentration, partial deactivation of active sites, or recombination of photogenerated electron–hole pairs, phenomena frequently reported in heterogeneous photocatalysis [94].

Notably, near-complete photodegradation of both MB and MO is achieved when using the Fe_3_O_4_- and ZnTiO_3_/TiO_2_-modified geopolymers, demonstrating the strong synergistic effect between the geopolymeric matrix and the embedded photocatalysts. The geopolymer structure likely acts as a stable support that facilitates dispersion of the active phases, enhances light accessibility, and promotes interaction between photogenerated reactive species and dye molecules. In contrast, the unmodified geopolymer exhibits negligible photocatalytic activity, with dye removal mainly attributed to photolysis rather than true photocatalytic degradation. This observation confirms that the aluminosilicate geopolymer matrix alone does not significantly contribute to photoinduced oxidation processes under UV irradiation. Therefore, the incorporation of Fe_3_O_4_ and ZnTiO_3_/TiO_2_ is essential to impart photocatalytic functionality to the geopolymeric materials.

### 4.6. Proposed Adsorption–Photocatalytic Mechanism

**Figure 11:**
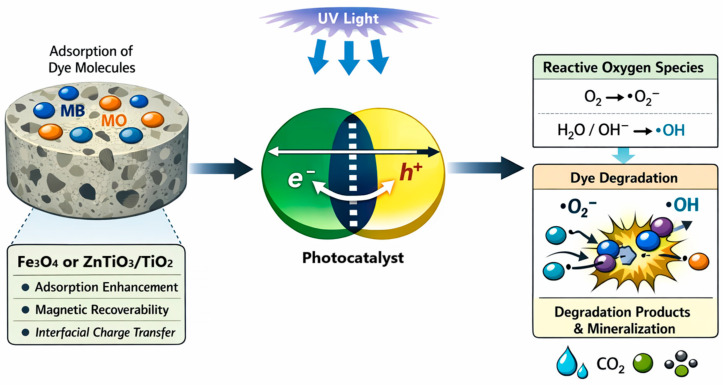
Mechanism of adsorption and photodegradation of dyes using Fe_3_O_4_ or ZnTiO_3_/TiO_2_ nanoparticles supported on rice husk ash geopolymer.

A schematic mechanism is proposed in Figure 11 to explain the improved dye removal observed for the modified geopolymers. In this mechanism, the geopolymer matrix first adsorbs dye molecules and concentrates them near the active nanophases. Under UV irradiation, the photocatalyst generates electron–hole pairs, whose separation is promoted by the semiconductor interface. The resulting electrons and holes react with dissolved oxygen and surface hydroxyl groups to form reactive oxygen species, mainly •O_2_^−^ and •OH, which oxidatively degrade the adsorbed dye molecules. This coupled adsorption–photocatalysis process explains the superior performance of the modified materials compared with the unmodified geopolymer.

Figure 11 clearly shows that photocatalytic activity occurs under illumination. In this environment, electrons in the photocatalyst can be excited and then immediately transferred from the valence band (VB) to the conduction band (CB), generating an electron–hole pair (e−/h+) and leaving a hole (h+) in the VB (reaction (14)). The electron–hole pairs can recombine immediately (reaction (15)); some of them can also migrate to the catalyst surface and react separately with other species adsorbed on the surface, such as H_2_O, OH−, O_2_, and other molecules (R), such as MB and MO dyes. The holes in the semiconductor VB can oxidize adsorbed water or hydroxyl ions to form highly reactive hydroxyl radicals (reactions (16) and (17)). On the other hand, the electrons generated in the CB can react with adsorbed oxygen molecules to produce OH radicals through a series of reactions (reactions (18)–(21)). These hydroxyl radicals have a high capacity to degrade organic dyes such as MB and MO (reaction (22)). Furthermore, direct oxidation of these dyes could also occur via reaction with holes (reaction (23)) [95]. The following reactions represent the likely photodegradation mechanism of MB and MO on the surfaces of the modified geopolymers.

(14) $$\left(semiconductor\right)\overset{hv}{\to }semiconductor+e_{CB}^{-}+h_{VB}^{+}$$

(15) $$e_{CB}^{-}+h_{vb}^{+}\to heat$$

(16) $$H_{2}O_{ads}+h_{VB}^{+}⇌{\left(H^{+}+{OH}^{-}\right)}_{ads}+h_{VB}^{+}\to {OH}_{ads}^{•}$$

(17) $${OH}_{ads}^{-}+h_{VB}^{+}\to {OH}_{ads}^{•}$$

(18) $${\left(O_{2}\right)}_{ads}+e_{BC}^{-}\to O_{2}^{•-}$$

(19) $$O_{2}^{•-}+H^{+}\to {HO}_{2}^{•}$$

(20) $${2HO}_{2}^{•}\to H_{2}O_{2}+O_{2}$$

(21) $$H_{2}O_{2}+e^{-}\to {OH}^{•}+{OH}^{-}$$

(22) $$R+{OH}_{ads}^{•}\to R_{ads}^{\prime •}+H_{2}O\to degradation\;products$$

(23) $$R_{ads}+h_{VB}^{+}\to R_{ads}^{•+}\to degradation\;products$$

### 4.7. Evaluation of Efficiency and Reuse

The comparative analysis of adsorption and photocatalytic efficiencies highlights the dual functionality of the synthesized geopolymeric materials. The adsorption results show that all samples exhibit a comparable capacity to remove both methylene blue and methyl orange through adsorption alone, with efficiencies consistently around 55–65%. This behavior reflects the presence of accessible surface functional groups within the geopolymeric matrix capable of interacting with both cationic and anionic dyes, regardless of surface modification. Differences in adsorption efficiency among the samples are relatively modest, suggesting that the incorporation of Fe_3_O_4_ or ZnTiO_3_/TiO_2_ does not drastically alter the overall availability of adsorption sites. However, slight variations between MB and MO adsorption indicate that surface chemistry and dye structure influence the strength and nature of adsorbate–adsorbent interactions. In contrast, the photocatalytic degradation results reveal a pronounced effect of geopolymer modification. The negligible photodegradation observed for the unmodified geopolymer confirms that the aluminosilicate matrix alone does not exhibit significant photocatalytic activity under UV irradiation, and that dye removal in this case is dominated by adsorption or photolysis. The substantial increase in photodegradation efficiency observed for the Fe_3_O_4_- and ZnTiO_3_/TiO_2_-modified geopolymers demonstrates the crucial role of the embedded semiconductor phases in generating reactive species capable of oxidizing the dye molecules.

Among the modified materials, the ZnTiO_3_/TiO_2_-modified geopolymer consistently exhibits the highest photocatalytic efficiency for both dyes. This behavior can be attributed to the synergistic effect between ZnTiO_3_ and TiO_2_, which enhances charge separation and reduces electron–hole recombination, thereby increasing the availability of reactive radicals. The Fe_3_O_4_-modified geopolymer also shows high photocatalytic activity, although slightly lower than that of the ZnTiO_3_/TiO_2_ system, indicating effective but less efficient photoinduced oxidation.

The combined adsorption and photocatalytic results suggest that dye removal by the modified geopolymers proceeds through a sequential mechanism, where initial adsorption concentrates the dye molecules near the active photocatalytic sites, followed by efficient photodegradation under UV irradiation. This synergistic behavior enhances overall removal efficiency and supports the suitability of Fe_3_O_4_- and ZnTiO_3_/TiO_2_-modified geopolymers as multifunctional materials for wastewater treatment applications involving both cationic and anionic dyes.

Finally, the reusability results obtained under combined adsorption–photodegradation conditions provide valuable insight into the long-term stability and practical applicability of the geopolymeric materials. The gradual decline in removal efficiency observed over successive cycles can be attributed to partial loss of active adsorption sites, incomplete regeneration of the surface, and possible deactivation of photocatalytic sites during repeated UV exposure. Importantly, no evidence of catalyst leaching was detected throughout the reuse experiments. Atomic absorption spectroscopy (AAS) analysis performed on the supernatant solutions collected after each treatment cycle showed no measurable release of Fe, Zn, or Ti species into the aqueous phase, confirming the structural stability of the incorporated nanophases and their effective immobilization within the geopolymeric matrix.

The significantly higher and more stable removal efficiencies exhibited by the Fe_3_O_4_- and ZnTiO_3_/TiO_2_-modified geopolymers highlight the synergistic effect between adsorption and photocatalysis. During each cycle, adsorption concentrates dye molecules at or near the surface, facilitating their subsequent photocatalytic degradation. This process contributes to partial regeneration of adsorption sites, which explains why the modified geopolymers retain higher performance compared to the unmodified material. In contrast, the unmodified geopolymer relies primarily on adsorption for dye removal, with minimal contribution from photocatalytic degradation. As a result, active sites become progressively saturated over successive cycles, leading to a more pronounced decrease in removal efficiency. The lack of an effective photocatalytic regeneration mechanism limits the reusability of the unmodified geopolymer.

Among the modified materials, M3 consistently exhibits slightly higher removal efficiencies than M2 across all cycles, suggesting that the ZnTiO_3_/TiO_2_ system provides more effective photocatalytic regeneration and stability under repeated use. This behavior is consistent with the higher photocatalytic activity previously observed for M3 and supports its suitability for repeated treatment applications.

### 4.8. Comparative Performance Analysis with Reported Adsorbents and Photocatalysts

To evaluate the performance of the geopolymeric materials synthesized in this study, the adsorption capacities and photocatalytic degradation efficiencies obtained for methylene blue (MB) and methyl orange (MO) were compared with those reported in the literature for various adsorbent and photocatalytic systems.

Table 8 presents a comparison of adsorption capacities reported for different materials used in dye removal. The adsorption capacity of the geopolymer synthesized in this work (M1) reached 79.4 mg g^−1^ for methylene blue, which is higher than several conventional mineral adsorbents such as zeolites (21.4 mg g^−1^), modified bagasse fly ash (16.59 mg g^−1^), and graphene oxide/kaolin composites (28.01 mg g^−1^). Although some advanced carbon-based materials show significantly higher adsorption capacities, such as GO-based composites or nanoparticle-doped activated carbons, these materials generally require more complex synthesis procedures or higher-cost precursors.

The incorporation of metal oxide nanoparticles into the geopolymer matrix also influenced adsorption performance. In the present study, the ZnTiO_3_–TiO_2_ modified geopolymer (M3) exhibited an adsorption capacity of 60.73 mg g^−1^ for methylene blue, while the Fe_3_O_4_-modified geopolymer (M2) showed 57.4 mg g^−1^. These values remain comparable to those reported for several supported metal oxide systems and confirm that the geopolymer matrix provides a suitable porous structure for dye adsorption.

For methyl orange, the Fe_3_O_4_-modified geopolymer showed the highest adsorption capacity (88.02 mg g^−1^) among the synthesized materials. Although some carbon-based adsorbents reported in the literature exhibit higher adsorption capacities, the geopolymeric materials present advantages such as lower cost, simple preparation routes, and the possibility of combining adsorption and photocatalytic degradation in a single material.

A comparison of photocatalytic degradation efficiencies reported in the literature is presented in Table 9. As expected, the unmodified geopolymer (M1) exhibited negligible photocatalytic activity, with degradation efficiencies of approximately 4% for MB and 3% for MO, confirming that the geopolymer matrix mainly acts as a support material rather than an active photocatalyst. In contrast, the incorporation of semiconductor nanoparticles significantly improved the photocatalytic performance of the materials. The ZnTiO_3_/TiO_2_-modified geopolymer (M3) achieved 94% degradation of methylene blue under UV irradiation in 195 min, which is comparable to several supported photocatalysts reported in the literature. For example, fly ash-based geopolymer photocatalysts have been reported to reach 92.7% MB removal after 240 min, while perlite-based geopolymers achieved 97.9% degradation under UV irradiation. Similarly, TiO_2_–Fe_3_O_4_/bentonite composites reported in the literature exhibit around 90% removal efficiency in 90 min.

Several advanced nanocomposites exhibit slightly higher efficiencies, particularly those based on doped TiO_2_ systems or multi-component photocatalysts. For instance, C–TiO_2_–SnO_2_/coal fly ash composites have been reported to reach 99.25% MB removal under visible light, while bentonite/PDA/Fe_3_O_4_@CuO systems achieve approximately 99% degradation in 90 min. However, many of these materials involve more complex synthesis procedures or multi-step functionalization processes.

A similar trend is observed for methyl orange degradation. The ZnTiO_3_/TiO_2_-modified geopolymer (M3) achieved 92% removal efficiency under UV irradiation, which is comparable to values reported for several semiconductor-supported photocatalysts such as TiO_2_/porous ceramic (89.5%), ZnO/g-C_3_N_4_ (91%), and CoFe_2_O_4_–SiO_2_–TiO_2_ (93.46%). In contrast, some highly optimized photocatalytic systems such as Ag_3_PO_4_/bentonite or F-Si-TiO_2_/activated carbon can reach degradation efficiencies close to 97–99%, although these systems often involve noble metals or complex doping strategies.

Overall, the results demonstrate that the combination of adsorption and photocatalytic processes in geopolymer-supported nanomaterials represents an effective approach for dye removal. Although some advanced nanocomposites reported in the literature exhibit higher adsorption capacities or faster degradation rates, the materials developed in this work offer a balanced performance together with advantages such as low-cost precursors, structural stability, and the possibility of producing recyclable pelletized systems suitable for practical wastewater treatment applications.

## 5. Conclusions

This work demonstrates that Fe_3_O_4_ and ZnTiO_3_/TiO_2_ nanoparticles can be successfully integrated into a rice husk ash-based geopolymeric matrix to produce hybrid nanocomposites with multifunctional behavior. Structural analyses confirmed that alkaline activation generated a predominantly amorphous aluminosilicate framework, while XRD results evidenced the presence of nanocrystalline Fe_3_O_4_ and ZnTiO_3_/TiO_2_ phases without disruption of the geopolymeric network. SEM observations revealed differences in nanoparticle dispersion and agglomeration, indicating that the type of nanophase influences the resulting mesoscopic organization of the composites.

Surface modification affected textural properties and adsorption performance. Dye uptake followed the Langmuir isotherm and pseudo-second-order kinetics, with spontaneous and exothermic thermodynamic behavior. The Fe_3_O_4_-modified material enhanced methyl orange adsorption, whereas the unmodified geopolymer showed competitive capacity toward methylene blue. Under UV irradiation, the ZnTiO_3_/TiO_2_-modified composite achieved the highest photodegradation efficiencies, demonstrating the contribution of the semiconductor heterostructure to photocatalytic activity.

Although pelletization reduced specific surface area, the materials maintained functional performance and structural stability during reuse cycles. Overall, these findings establish geopolymeric matrices as effective nano-structured hosts for magnetic and semiconductor nanoparticles, enabling synergistic adsorption–photodegradation through controlled nanoparticle incorporation and preserved matrix integrity.

### Limitations and Future Perspectives

While this study demonstrates the potential of rice husk ash-based geopolymers modified with Fe_3_O_4_ and ZnTiO_3_/TiO_2_ nanoparticles for dye removal via adsorption and photocatalytic processes, several limitations should be acknowledged. First, although the structural and morphological characterization performed in this study confirmed the successful incorporation of the modifying nanophases, additional techniques such as UV-DRS, photoluminescence spectroscopy, and transmission electron microscopy could provide a greater understanding of the optical properties and nanoscale structure of the semiconductor components. Second, the recyclability tests performed in this work were conceived as a preliminary assessment of the material’s stability during repeated treatment cycles. A more complete understanding of the regeneration mechanisms would require further structural and textural analyses of the reused materials using techniques such as XRD, BET, and FTIR after multiple cycles. Therefore, future studies will focus on a deeper investigation of regeneration mechanisms, long-term stability, and advanced characterization of hybrid materials to better understand the relationship between structural properties and photocatalytic performance.

## Figures and Tables

**Table 1:** Composition of geopolymer samples.

Compound	M1	M2	M3
NaOH	0.8 g	0.8 g	0.8 g
Na_2_SiO_3_	4.4 g	4.4 g	4.4 g
H_2_O	1.5 mL	1.5 mL	1.5 mL
CH_3_(CH_2_)_11_OSO_3_Na	0.5 g	0.5 g	0.5 g
H_2_O_2_	0.5 mL	0.5 mL	0.5 mL
MK	3.75 g	3.75 g	3.75 g
RHA	1.25 g	1.25 g	1.25 g
Fe_3_O_4_	-	1 g	-
ZnTiO_3_/TiO_2_	-	-	1 g

**Table 2:** Chemical composition of rice husk and geopolymers.

	RHA	M1	M2	M3
MgO (%)	3.36 (±1.02)	4.13 (±0.95)	3.73 (±0.86)	3.36 (±1.01)
Al_2_O_3_ (%)	2.64 (±0.12)	23.41 (±1.18)	21.23 (±1.82)	21.81 (±1.73)
SiO_2_ (%)	6.37 (±1.11)	42.10 (±2.14)	39.71 (±2.56)	40.39 (±2.66)
P_2_O_5_ (%)	5.31 (±1.05)	3.81 (±1.06)	3.77 (±1.14)	3.31 (±1.09)
K_2_O (%)	2.76 (±0.07)	1.31 (±0.17)	1.27 (±0.22)	1.29 (±0.19)
CaO (%)	0.34 (±0.02)	0.28 (±0.06)	0.14 (±0.05)	0.18 (±0.05)
TiO_2_ (%)	0.04 (±0.01)	0.46 (±0.02)	0.37 (±0.03)	6.13 (±1.29)
Fe_2_O_3_ (%)	0.18 (±0.05)	0.28 (±0.09)	8.22 (±1.57)	0.28 (±0.09)
ZnO (%)	0.02 (±0.01)	0.03 (±0.01)	0.04 (±0.02)	1.99 (±0.06)

**Table 3:** Elemental analysis (wt%) of geopolymer, Fe_3_O_4_ modified geopolymer and ZnTiO_3_/TiO_2_ modified geopolymer.

Samples	C	O	Na	Al	Si	K	Ca	Fe	Ti	Zn
M1	8.4	46.2	1.6	10.4	27.6	1.9	2.8	0.7	-	-
M2	7.6	42.8	-	8.6	18.2	1.3	-	18.5	0.8	-
M3	7.4	47.3	-	8.1	17.1	1.3	-	0.3	13.7	4.8

**Table 4:** Specific Surface Area (SSA) and Monolayer Volume (MV) of Geopolymer, Fe_3_O_4_ modified geopolymer and ZnTiO_3_/TiO_2_ modified geopolymer.

Samples	SSA (m^2^ g^−1^)	MV (m^3^ g^−1^)
Geopolymer (powder)	109	25.7
Geopolymer (pellets)	85.2	22.4
Fe_3_O_4_ modified geopolymer (powder)	190	40.9
Fe_3_O_4_ modified geopolymer (pellets)	24.5	5.88
ZnTiO_3_/TiO_2_ modified geopolymer (powder)	198	41.0
ZnTiO_3_/TiO_2_ modified geopolymer (pellets)	31.6	6.20

**Table 5:** Isotherm parameters for methylene blue and methyl orange adsorption on the geopolymers.

Isotherm Parameters		Methyl Blue (MB)			Methyl Orange (MO)		
		M1	M2	M3	M1	M2	M3
Langmuir	q_max_ (mg g^−1^)	79.40	57.76	60.73	55.28	88.10	53.52
		(±1.97)	(±2.33)	(±1.23)	(±1.47)	(±1.63)	(±1.30)
	K_L_ (L mg^−1^)	1.53	2.41	1.51	0.87	1.51	1.41
		(±0.14)	(±0.16)	(±0.15)	(±0.09)	(±0.11)	(±0.13)
	R_L_	0.02	0.01	0.02	0.04	0.02	0.02
	R^2^	1.00	1.00	1.00	1.00	1.00	0.99
Freundlich	K_F_ (mg g^−1^)	4.38	4.12	3.97	3.73	5.30	3.68
		(±0.94)	(±1.55)	(±1.48)	(±1.22)	(±1.35)	(±1.36)
	n	1.83	2.00	1.82	1.48	1.43	1.98
		(±0.29)	(±0.34)	(±0.33)	(±0.29)	(±0.31)	(±0.33)
	R^2^	0.96	0.97	0.97	0.97	0.99	0.94

Experimental conditions: pH = 7, v: 0.5 L, w: 0.05 g, T: 293.15 K.

**Table 6:** Thermodynamic parameters for methylene blue and methyl orange adsorption on M1, M2 and M3 geopolymers at three temperatures.

System	Compounds	Temperature (K)	ln k_C_	∆G° (kJ mol^−1^)	∆H° (kJ mol^−1^)	∆S° (kJ mol^−1^ K^−1^)
Methyl Blue	M1	293.15	17.12	−41.72	−9.66	0.18
		298.15	17.16	−42.54		
		303.15	17.25	−43.47		
	M2	293.15	17.10	−41.68	−10.91	0.18
		298.15	17.17	−42.57		
		303.15	17.25	−43.47		
	M3	293.15	17.11	−41.69	−13.96	0.19
		298.15	17.23	−42.72		
		303.15	17.30	−43.59		
Methyl Orange	M1	293.15	17.19	−41.89	−10.52	0.18
		298.15	17.25	−42.75		
		303.15	17.33	−43.68		
	M2	293.15	17.10	−41.69	−12.97	0.19
		298.15	17.19	−42.60		
		303.15	17.28	−43.55		
	M3	293.15	17.04	−41.53	−14.40	0.19
		298.15	17.17	−42.56		
		303.15	17.23	−43.43		

Experimental conditions: C_0_ MB and MO: 20 mg L^−1^; v: 0.5 L, w: 0.05 g, pH = 7.

**Table 7:** Kinetic parameters for methylene blue and methyl orange adsorption on the geopolymers.

Kinetic Parameters		Methyl Blue (MB)			Methyl Orange (MO)		
		M1	M2	M3	M1	M2	M3
Pseudo-first order	q_e_ (mg g^−1^)	166.82	159.32	161.01	155.01	170.50	111.75
		(±3.15)	(±2.87)	(±3.35)	(±2.42)	(±4.04)	(±2.73)
	K_1_ (h^−1^)	0.04	0.03	0.04	0.03	0.03	0.03
		(±3.38 × 10^−03^)	(±6.75 × 10^−03^)	(±4.10 × 10^−03^)	(±4.09 × 10^−03^)	(±4.83 × 10^−03^)	(±4.75 × 10^−03^)
	R^2^	1.00	0.99	1.00	0.98	0.98	0.94
Pseudo-second order	q_e_ (mg g^−1^)	216.25	211.07	212.60	210.96	214.50	204.80
		(±2.20)	(±2.84)	(±2.41)	(±2.44)	(±1.48)	(±2.64)
	K_2_ (g mg^−1^ h^−1^)	3.48 × 10^−04^	3.05 × 10^−04^	3.85 × 10^−04^	3.29 × 10^−04^	3.12 × 10^−04^	5.06 × 10^−04^
		(±7.29 × 10^−05^)	(±7.57 × 10^−05^)	(±7.31 × 10^−05^)	(±8.09 × 10^−05^)	(±7.97 × 10^−05^)	(±6.82 × 10^−05^)
	R^2^	1.00	1.00	1.00	1.00	1.00	1.00
Intraparticle diffusion	D_f_ (m^2^ min^−1^)	3.19 × 10^−11^	2.73 × 10^−11^	2.98 × 10^−11^	2.82 × 10^−11^	3.16 × 10^−11^	3.11 × 10^−11^
	R^2^	0.99	0.99	0.99	0.98	0.98	0.99
	D_p_ (m^2^ min^−1^)	1.40 × 10^−17^	1.20 × 10^−17^	1.30 × 10^−17^	1.30 × 10^−17^	1.40 × 10^−17^	1.40 × 10^−17^
	R^2^	0.99	1.00	0.99	0.99	0.97	0.99
	K_d1_ (g mg^−1^ h^−1^)	22.58	22.53	22.47	22.61	22.56	23.50
		(±0.80)	(±0.85)	(±0.84)	(±0.66)	(±0.76)	(±0.59)
	R^2^	0.99	0.99	0.99	0.99	0.99	0.95
	K_d2_ (g mg^−1^ h^−1^)	2.70	2.95	1.87	2.38	2.87	1.50
		(±0.14)	(±0.10)	(±0.15)	(±0.10)	(±0.13)	(±0.11)
	R^2^	0.82	0.95	0.82	0.94	0.90	0.88

Experimental conditions: C_0_ MB and MO: 20 mg L^−1^; v: 0.5 L, pH = 7, w: 0.05 g, T: 293.15 K.

**Table 8:** Comparison of adsorption capacities (q_e_) for methylene blue (MB) and methyl orange (MO) using geopolymeric materials developed in this study and other adsorbents reported in the literature.

Adsorbent Material	Dye	Adsorption Capacity, q_e_ (mg g^−1^)	Ref.
Geopolymer	MB	48.97	[96]
ZnTiO_3_–TiO_2_/Geopolymer	MB	61.96	[96]
TiO_2_–Cu_x_S/fly ash	MB	0.36	[97]
GO/DSAC	MB	667	[98]
CSAC@AgNPs@TiO_2_NPs	MB	186.3	[99]
FeMn/HNTs	MB	96.47	[100]
TiO_2_/PVA	MB	138.88	[101]
GO/Kaolin	MB	28.01	[102]
Kaolinite	MB	52.76	[103]
Zeolite	MB	21.4	[104]
Modified bagasse fly ash	MB	16.59	[105]
Geopolymer (M1)	MB	79.4	This study
Fe_3_O_4_-modified geopolymer (M2)	MB	57.4	This study
ZnTiO_3_–TiO_2_-modified geopolymer (M3)	MB	60.73	This study
PIL–GO/TiO_2_/Fe_3_O_4_	MO	67.88	[106]
Ag_3_PO_4_/Bentonite	MO	98	[107]
Fe_2_O_3_/Activated carbon	MO	362	[108]
Fe_3_O_4_/Activated carbon	MO	150.4	[109]
Ag-N-ZnO/Coconut husk activated carbon	MO	90.42	[110]
Fe_2_O_3_/Mesoporous carbon	MO	69	[108]
Fe_2_O_3_/Biochar	MO	16.05	[111]
α-Fe_2_O_3_ nanoparticles	MO	28.9	[112]
Magnetic lignin-based carbon nanoparticles	MO	113	[113]
Fe_3_O_4_/Polypyrrole	MO	149.5	[114]
Chitosan/Bentonite	MO	136.8	[115]
CuO nanoparticles	MO	217.4	[116]
Fe_3_O_4_/Graphene oxide	MO	714.3	[117]
Geopolymer (M1)	MO	55.58	This study
Fe_3_O_4_-modified geopolymer (M2)	MO	88.02	This study
ZnTiO_3_–TiO_2_-modified geopolymer (M3)	MO	53.52	This study

**Table 9:** Comparison of photocatalytic degradation efficiencies of methylene blue (MB) and methyl orange (MO) using different supported nanomaterials reported in the literature and the geopolymer-based photocatalysts developed in this study.

Photocatalyst	Dye	C_0_ (mg L^−1^)	Light Source	Time (min)	Removal Efficiency (%)	Ref.
Geopolymer	MB	20	UV irradiation	150	4	[96]
Geopolymer/ZnTiO_3_–TiO_2_	MB	20	UV irradiation	150	93	[96]
Fly ash-based geopolymer	MB	15	UV irradiation	240	92.7	[118]
TiO_2_-doped zeolite/geopolymer	MB	40	UV irradiation	180	99.1	[119]
Zeolite/geopolymer	MB	40	UV irradiation	180	92.5	[119]
Bentonite/PDA/Fe_3_O_4_@CuO	MB	10	UV irradiation	90	99	[120]
TiO_2_/zeolite/coal fly ash	MB	–	UV irradiation + H_2_O_2_	60	99	[121]
C–TiO_2_–SnO_2_/coal fly ash	MB	20	Visible irradiation	180	99.25	[122]
TiO_2_–Cu_x_S/fly ash	MB	4	UV irradiation	360	96	[97]
TiO_2_–Fe_3_O_4_/bentonite	MB	30	Xenon lamp irradiation	90	90	[123]
Perlite-based geopolymer	MB	30	UV irradiation	240	97.9	[124]
TiO_2_-coated geopolymer spheres	MB	–	UV irradiation	600	93	[125]
Geopolymer (M1)	MB	20	UV irradiation	195	4	This study
Fe_3_O_4_-modified geopolymer (M2)	MB	20	UV irradiation	195	85	This study
ZnTiO_3_/TiO_2_-modified geopolymer (M3)	MB	20	UV irradiation	195	94	This study
C–TiO_2_–SnO_2_/coal fly ash	MO	20	Visible irradiation	180	97.75	[122]
Zn/bentonite	MO	15	UV irradiation	60	64.92	[126]
TiO_2_/bentonite	MO	3.27	UV irradiation	180	79.5	[127]
CoFe_2_O_4_–SiO_2_–TiO_2_	MO	25	UV irradiation	200	93.46	[128]
F-Si-TiO_2_/activated carbon	MO	–	Visible irradiation	70	97.7	[129]
TiO_2_/porous ceramic	MO	25	UV irradiation	120	89.5	[130]
PIL–GO/TiO_2_/Fe_3_O_4_	MO	30	UV irradiation	180	95	[106]
Ag_3_PO_4_/bentonite	MO	70	Visible irradiation	30	99	[107]
TiO_2_/zeolite	MO	30	UV irradiation	300	87	[131]
ZnO/g-C_3_N_4_	MO	3.27	Visible irradiation	120	91	[132]
NiFe_2_O_4_/SiO_2_/NiO	MO	10	UV irradiation	120	95.7	[133]
Geopolymer (M1)	MO	20	UV irradiation	195	3	This study
Fe_3_O_4_-modified geopolymer (M2)	MO	20	UV irradiation	195	82	This study
ZnTiO_3_/TiO_2_-modified geopolymer (M3)	MO	20	UV irradiation	195	92	This study

## Data Availability

Data are availability in the manuscript.

## Supplementary Materials

The following supporting information can be downloaded at: https://www.mdpi.com/article/10.3390/nano16070413/s1. Table S1. Equilibrium adsorption data of MB on geopolymer M1, Table S2. Equilibrium adsorption data of MB on geopolymer M2, Table S3. Equilibrium adsorption data of MB on geopolymer M3. Table S4. Equilibrium adsorption data of MO on geopolymer M1, Table S5. Equilibrium adsorption data of MO on geopolymer M2, Table S6. Equilibrium adsorption data of MO on geopolymer M3. Figure S1. Linear fit of the (a) Langmuir and (b) Freundlich isotherm models for the adsorption of MB on geopolymer M1. Figure S2. Linear fit of the (a) Langmuir and (b) Freundlich isotherm models for the adsorption of MB on geopolymer M2. Figure S3. Linear fit of the (a) Langmuir and (b) Freundlich isotherm models for the adsorption of MB on geopolymer M3. Figure S4. Linear fit of the (a) Langmuir and (b) Freundlich isotherm models for the adsorption of MO on geopolymer M1. Figure S5. Linear fit of the (a) Langmuir and (b) Freundlich isotherm models for the adsorption of MO on geopolymer M2. Figure S6. Linear fit of the (a) Langmuir and (b) Freundlich isotherm models for the adsorption of MO on geopolymer M3. Figure S7. Nitrogen adsorption–desorption curves recorded during BET surface area analysis based on the Brunauer–Emmett–Teller (BET) isotherm method for (a) M1 (powder), (b) M1 (pellets), (c) M2 (powder), (d) M2 (pellets), (e) M3 (powder), and (f) M3 (pellets).

## Abbreviations

The following abbreviations are used in this manuscript:

MK	Metakaolin
RHA	Rice Husk Ash
M1	Unmodified geopolymer
M2	Fe_3_O_4_-modified geopolymer
M3	ZnTiO_3_/TiO_2_-modified geopolymer
MB	Methyl Blue
MO	Methyl Orange
