# Supplementary material for: Rice Husk Ash Geopolymers Modified with Fe3O4 or ZnTiO3/TiO2 Nanoparticles for the Adsorption and Photodegradation of Organic Dyes
Source: Nanomaterials (Basel). 2026 Mar 29;16(7):413. doi: 10.3390/nano16070413 (PMC13075101; doi:10.3390/nano16070413)
Supplement: Supplementary file 1 [file nanomaterials-16-00413-s001.zip › nanomaterials-4183297-supplementary.pdf]

Supplementary Materials

Rice Husk Ash Geopolymers Modified with Fe_3O_4 or $ZnTiO_3/TiO_2$ Nanoparticles for the Adsorption and Photodegradation of Organic Dyes

Ximena Jaramillo-Fierro [1,*], Juan-Pablo Cueva [2], John Ramón [2] and Eduardo Valarezo [1]

[1] Departamento de Química, Facultad de Ciencias Exactas y Naturales, Universidad Técnica Particular de Loja, San Cayetano Alto, Loja 1101608, Ecuador

[2] Carrera de Ingeniería Química, Facultad de Ciencias Exactas y Naturales, Universidad Técnica Particular de Loja, San Cayetano Alto, Loja 1101608, Ecuador

* Correspondence: xvjaramillo@utpl.edu.ec; Tel.: +593-7-3701444

Table S1. Equilibrium adsorption data of MB on geopolymer M1.

Co (mg/L)	Ce (mg/L)	qe (mg/g)
30	19.59	104.089343
25	14.62	103.792788
20	10.00	100.045210
15	5.99	90.062809
10	2.66	73.387513
5	0.98	40.237890
2	0.23	17.681593
1	0.10	9.049408
0.5	0.04	4.618794
0.25	0.02	2.303948

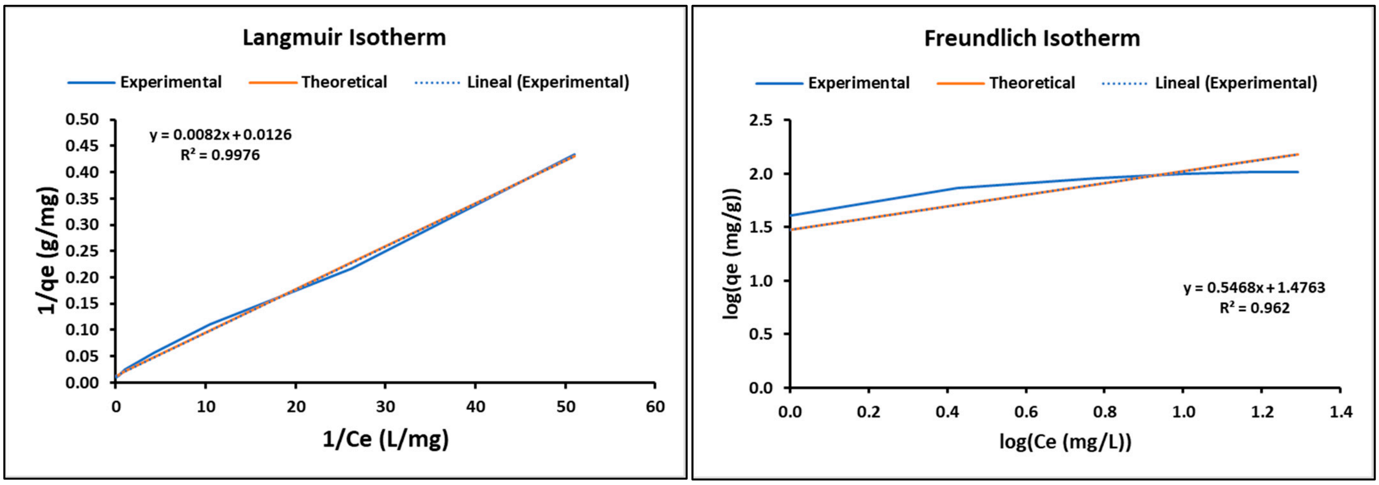

Figure S1. Linear fit of the (a) Langmuir and (b) Freundlich isotherm models for the adsorption of MB on geopolymer M1. The fit was performed using the linearized equations of each model, based on the equilibrium adsorption data. The corresponding R^2 values are presented for comparison of the models.

Table S2. Equilibrium adsorption data of MB on geopolymer M2.

Co (mg/L)	Ce (mg/L)	qe (mg/g)
30	21.12	88.751776
25	16.11	88.882670
20	11.44	85.631970
15	7.26	77.368461
10	4.10	59.020452
5	1.13	38.740797
2	0.26	17.400702
1	0.09	9.142084
0.5	0.04	4.617318
0.25	0.02	2.328642

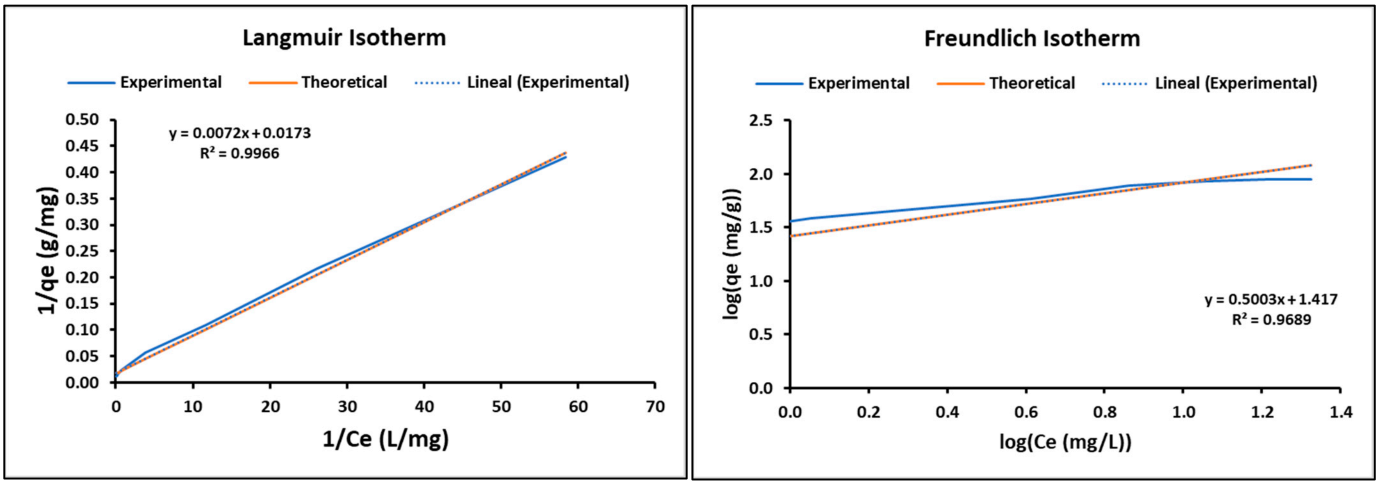

Figure S2. Linear fit of the (a) Langmuir and (b) Freundlich isotherm models for the adsorption of MB on geopolymer M2. The fit was performed using the linearized equations of each model, based on the equilibrium adsorption data. The corresponding R^2 values are presented for comparison of the models.

Table S3. Equilibrium adsorption data of MB on geopolymer M3.

Co (mg/L)	Ce (mg/L)	qe (mg/g)
30	20.93	90.705059
25	15.93	90.665231
20	11.21	87.891819
15	6.99	80.096878
10	3.93	60.721206
5	1.29	37.091496
2	0.36	16.380624
1	0.12	8.808934
0.5	0.06	4.447470
0.25	0.02	2.250129

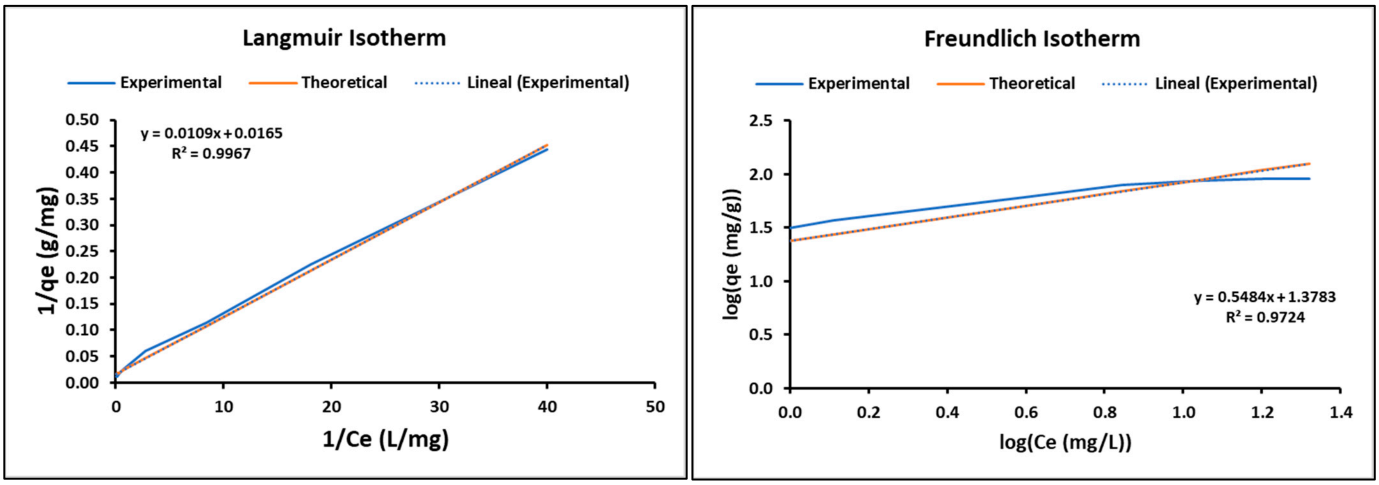

Figure S3. Linear fit of the (a) Langmuir and (b) Freundlich isotherm models for the adsorption of MB on geopolymer M3. The fit was performed using the linearized equations of each model, based on the equilibrium adsorption data. The corresponding R^2 values are presented for comparison of the models.

Table S4. Equilibrium adsorption data of MO on geopolymer M1.

Co (mg/L)	Ce (mg/L)	qe (mg/g)
30	19.63	103.717653
25	14.76	102.417115

20	10.17	98.272551
15	6.14	88.615501
10	3.15	68.503337
5	1.44	35.567234
2	0.56	14.361572
1	0.24	7.561572
0.5	0.10	3.994044
0.25	0.04	2.071051

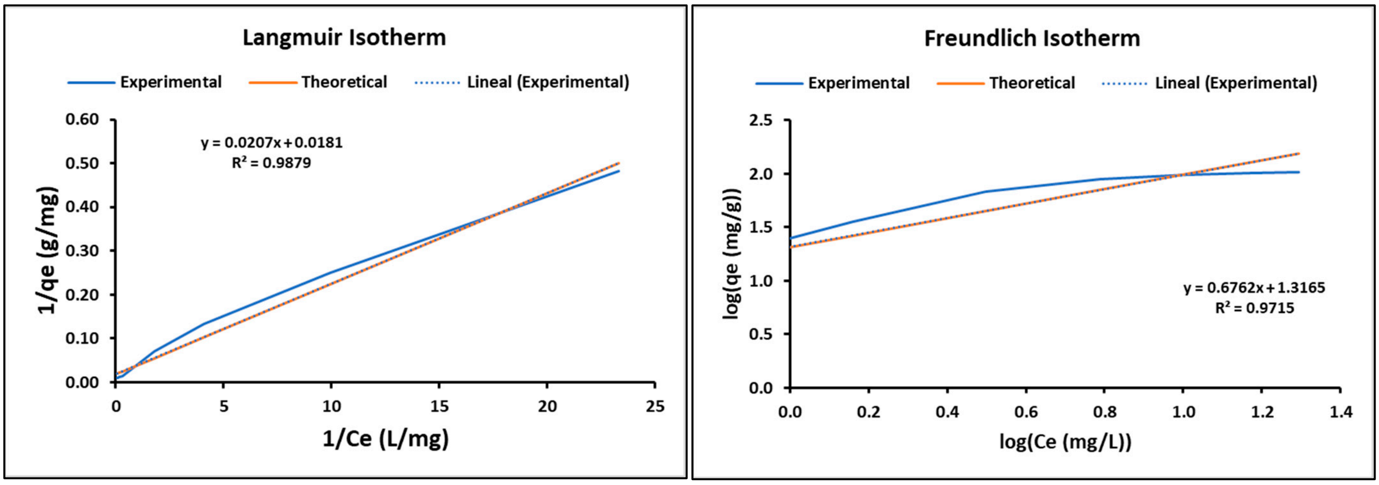

Figure S4. Linear fit of the (a) Langmuir and (b) Freundlich isotherm models for the adsorption of MO on geopolymer M1. The fit was performed using the linearized equations of each model, based on the equilibrium adsorption data. The corresponding R^2 values are presented for comparison of the models.

Table S5. Equilibrium adsorption data of MO on geopolymer M2.

Co (mg/L)	Ce (mg/L)	qe (mg/g)
30	10.97	190.253994
25	7.04	179.587549
20	4.73	152.693219
15	3.37	116.293864
10	1.90	80.970245
5	0.85	41.549927
2	0.21	17.925045
1	0.09	9.117812
0.5	0.04	4.612398
0.25	0.02	2.323961

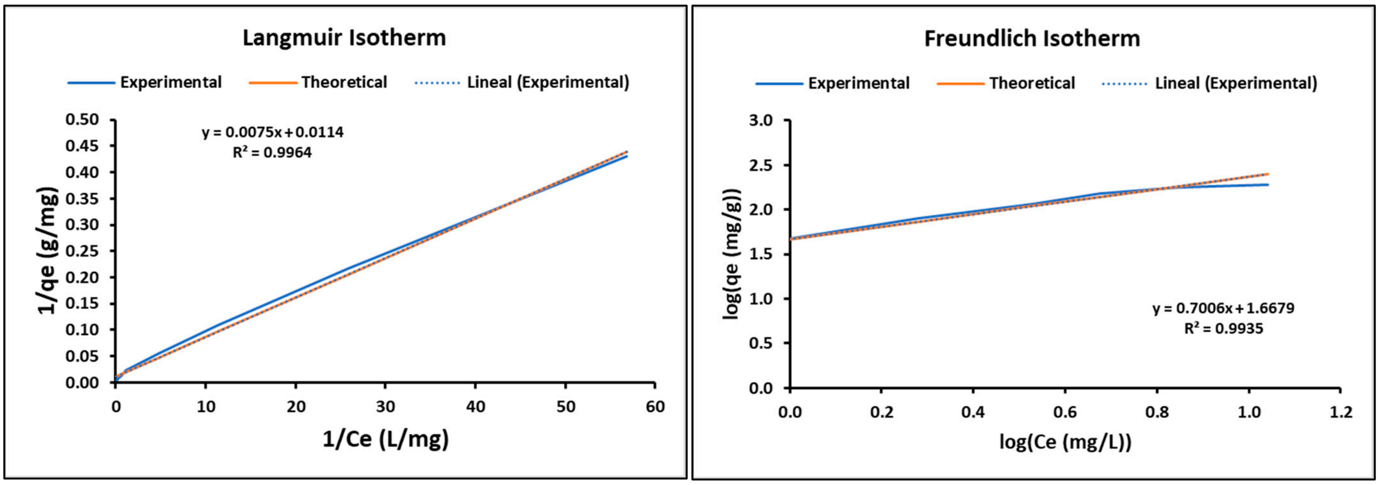

Figure S5. Linear fit of the (a) Langmuir and (b) Freundlich isotherm models for the adsorption of MO on geopolymer M2. The fit was performed using the linearized equations of each model, based on the equilibrium adsorption data. The corresponding R^2 values are presented for comparison of the models.

Table S6. Equilibrium adsorption data of MO on geopolymer M3.

Co (mg/L)	Ce (mg/L)	qe (mg/g)
30	22.92	70.844995
25	17.98	70.212056
20	13.18	68.166846
15	8.76	62.350915
10	4.72	52.833154
5	1.28	37.175457
2	0.30	17.030893
1	0.15	8.456082
0.5	0.07	4.289429
0.25	0.03	2.205576

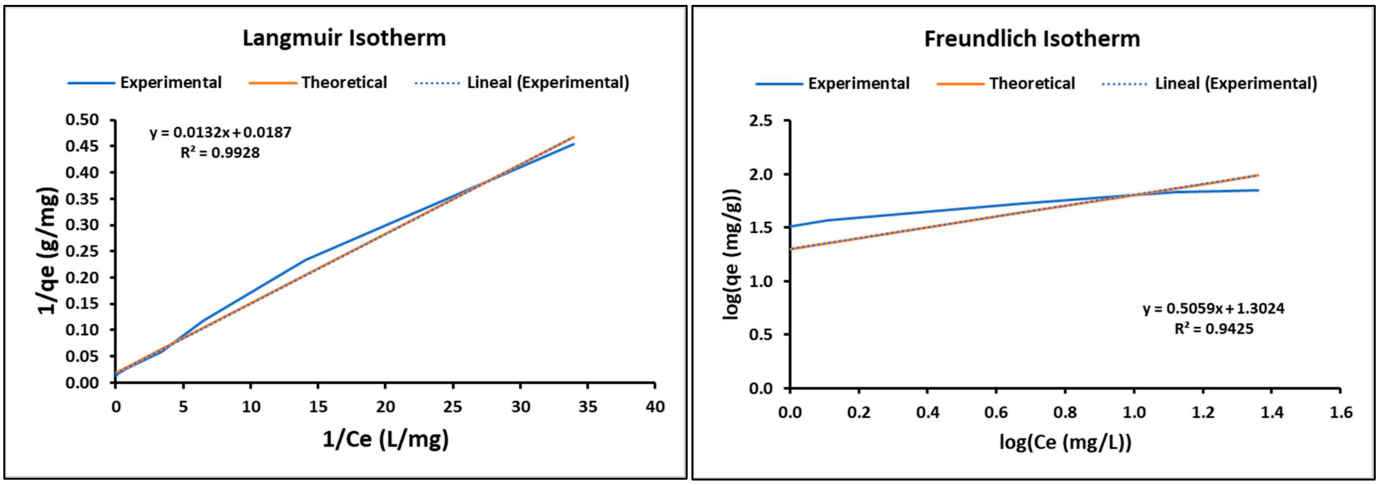

Figure S6 Linear fit of the (a) Langmuir and (b) Freundlich isotherm models for the adsorption of MO on geopolymer M3. The fit was performed using the linearized equations of each model, based on the equilibrium adsorption data. The corresponding R^2 values are presented for comparison of the models.

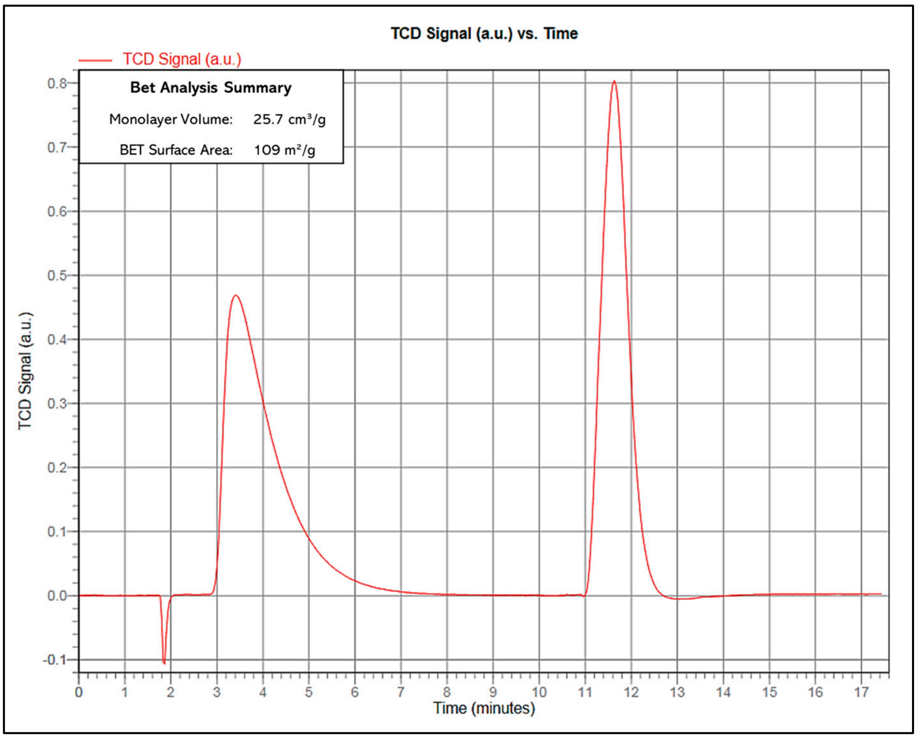

TCD Signal (a.u.) vs. Time
TCD Signal (a.u.)
Bet Analysis Summary
Monolayer Volume: 25.7 cm³/g
BET Surface Area: 109 m²/g
TCD Signal (a.u.)
Time (minutes)

(a)

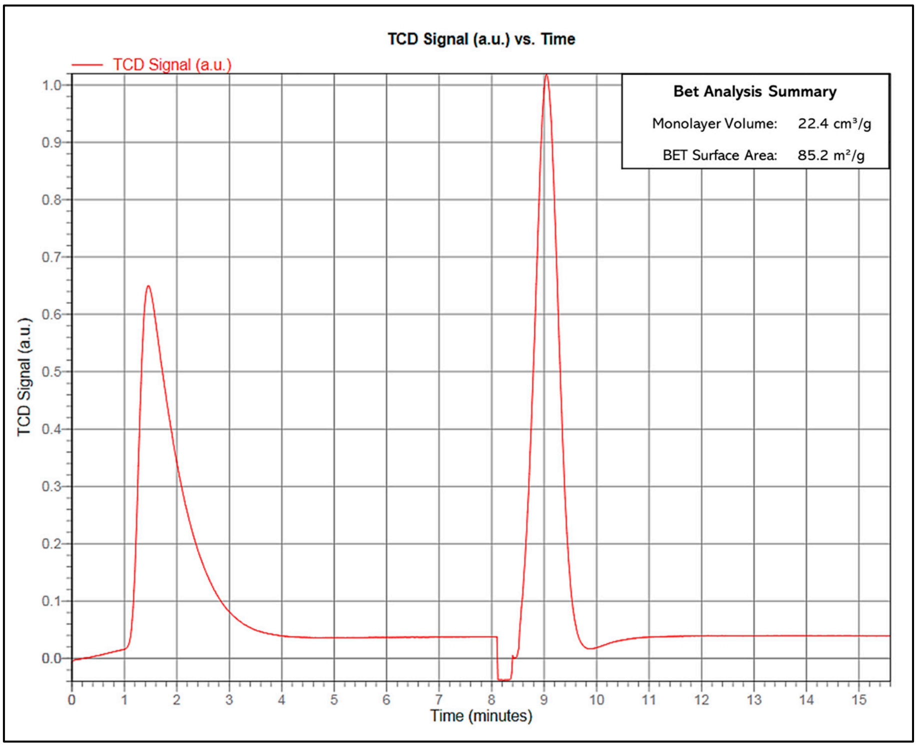

TCD Signal (a.u.) vs. Time
TCD Signal (a.u.)
Bet Analysis Summary
Monolayer Volume: 22.4 cm³/g
BET Surface Area: 85.2 m²/g
TCD Signal (a.u.)
Time (minutes)

b)

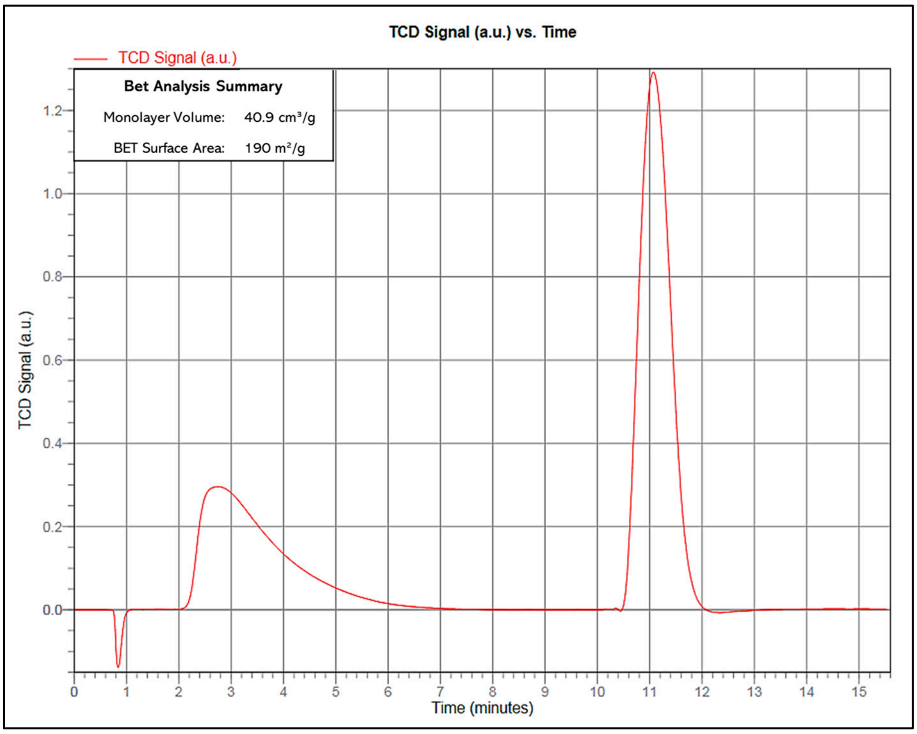
TCD Signal (a.u.) vs. Time
TCD Signal (a.u.)
Bet Analysis Summary
Monolayer Volume: 40.9 cm³/g
BET Surface Area: 190 m²/g
TCD Signal (a.u.)
Time (minutes)

(c)

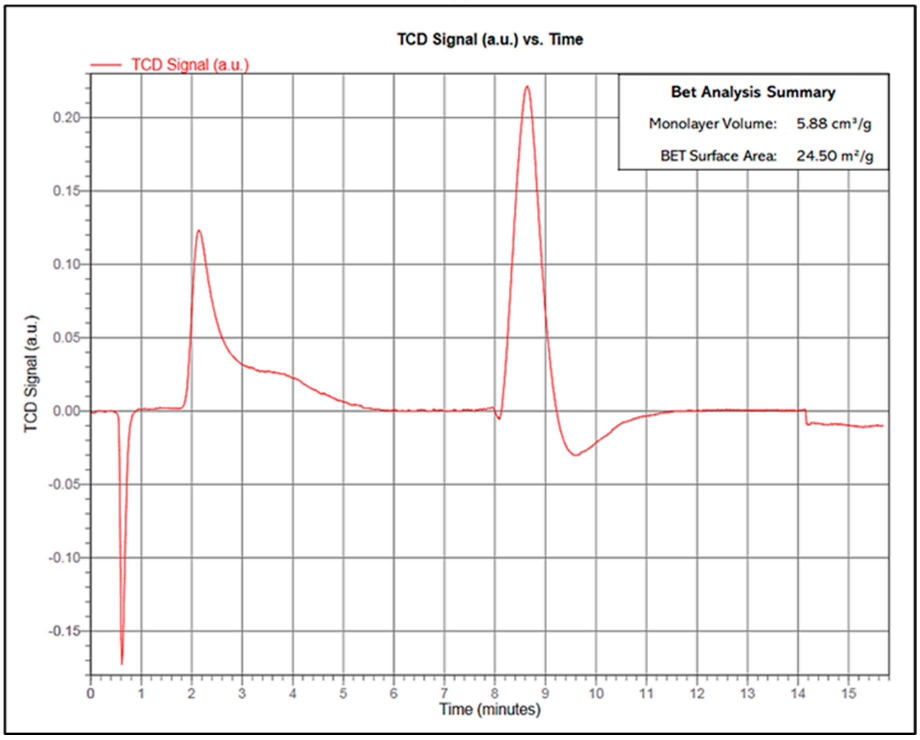
TCD Signal (a.u.) vs. Time
TCD Signal (a.u.)
Bet Analysis Summary
Monolayer Volume: 5.88 cm³/g
BET Surface Area: 24.50 m²/g
TCD Signal (a.u.)
Time (minutes)

(d)

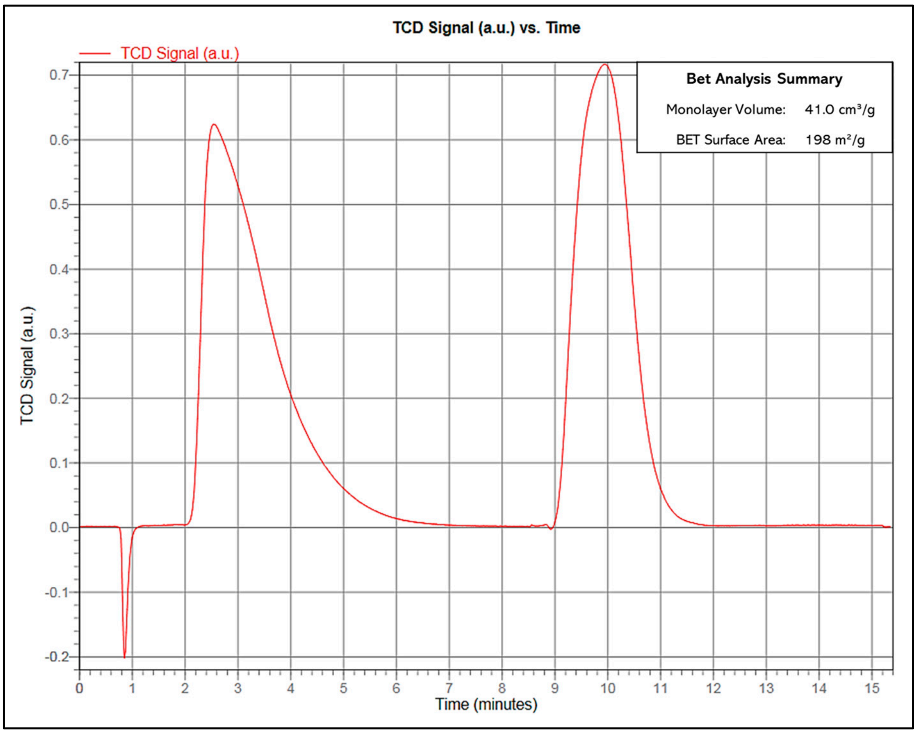

(e)

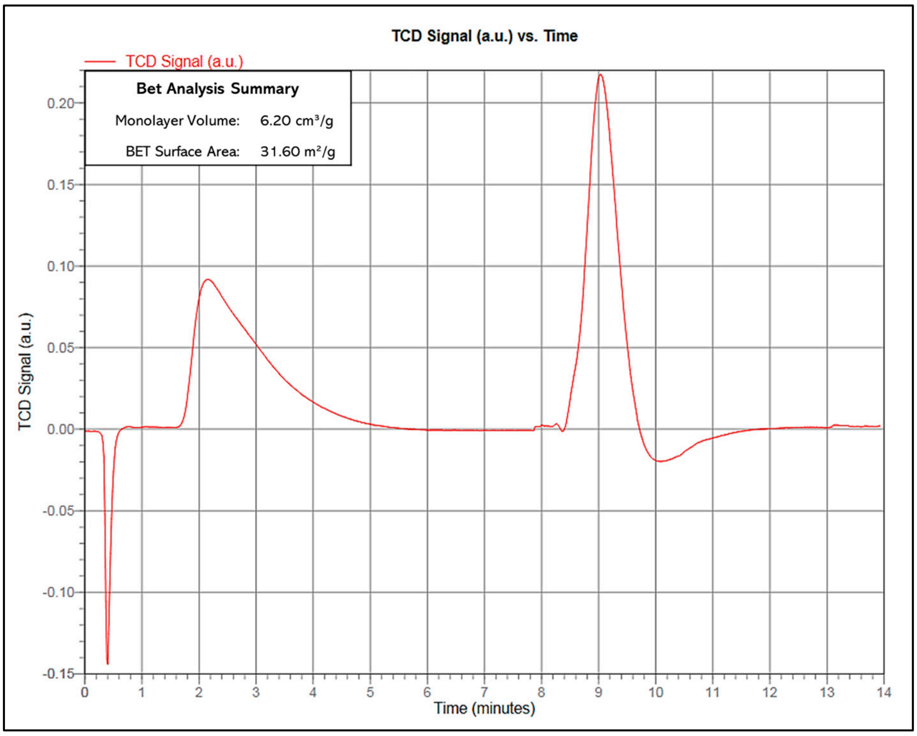

(f)

Figure S7. Nitrogen adsorption–desorption curves recorded during BET surface area analysis based on the Brunauer–Emmett–Teller (BET) isotherm method for (a) M1 (powder), (b) M1 (pellets), (c) M2 (powder), (d) M2 (pellets), (e) M3 (powder), and (f) M3 (pellets). The curves correspond to the detector signals obtained during the adsorption–desorption process from which the BET parameters (monolayer volume and specific surface area) were calculated.
